# Differential fracture risk among proton pump inhibitors in older adults: evidence network and pharmacovigilance validation

**DOI:** 10.3389/fphar.2026.1835602

**Published:** 2026-08-19

**Authors:** Hui-ling Piao, Zeng-hao Wu, Ling-yun Zhou, Chang-hong Lai, Zhi-yao Tang, Wen-jun Yin, Yi-hu Yi

**Affiliations:** 1 Department of Pharmacy, The Third Xiangya Hospital of Central South University, Changsha, Hunan, China; 2 Department of Orthopedics, The Second Xiangya Hospital, Central South University, Changsha, Hunan, China

**Keywords:** disproportionality analysis, FAERS database, fracture risk in the elderly, network meta-analysis, osteoporosis, pharmacovigilance, proton pump inhibitors

## Abstract

**Background:**

Proton pump inhibitors (PPIs) are the first-line therapy for acid-related disorders, with particularly high rates of chronic use in adults aged ≥65 years. Nearly all existing studies treat PPIs as a uniform drug class, overlooking potential heterogeneity in fracture risk across individual agents. This study aimed to evaluate the relative fracture risk of individual PPIs in elderly adults, using a complementary dual-method approach of network meta-analysis (NMA) and disproportionality analysis.

**Methods:**

First, we performed a NMA to compare the relative risk of any-site fracture associated with individual PPIs. Second, we performed a complementary disproportionality analysis using data from the US FDA Adverse Event Reporting System (FAERS) database, with additional analysis of osteoporosis/osteopenia events defined by the Standardised Medical Dictionary for Regulatory Activities (MedDRA) Standardised Query (SMQ).

**Results:**

The NMA included 9 eligible studies, enrolling a total of 257,445 participants. Compared with non-PPI users, omeprazole (OR 1.51, 95%CI 1.36–1.67), rabeprazole (OR 1.47, 95%CI 1.22–1.78), pantoprazole (OR 1.44, 95%CI 1.28–1.62), and lansoprazole (OR 1.32, 95%CI 1.14–1.53) were associated with a significantly increased risk of any-site fracture. In contrast, esomeprazole showed no significant association with overall fracture risk (OR 1.16, 95%CI 0.97–1.38). Pairwise comparisons from the NMA showed that esomeprazole had a lower relative fracture risk compared with other individual PPIs. In the FAERS analysis, we identified 16,908 PPI-related fracture adverse events. Consistent with NMA findings, omeprazole exhibited a significant fracture risk signal (ROR 1.25, 95%CI 1.03–1.51), while esomeprazole showed no significant signal for overall fracture (ROR 0.89, 95%CI 0.66–1.20). However, esomeprazole presented a strong positive risk signal in the narrow-scope MedDRA SMQ analysis for Osteoporosis/Osteopenia (ROR 2.44, 95%CI 1.41–4.20).

**Conclusion:**

Long-term PPI use is associated with increased fracture risk in elderly adults, with significant heterogeneity in bone safety profiles across individual PPI agents. Although esomeprazole was not significantly associated with fracture risk in NMA or FAERS fracture analyses, it showed a strong narrow-scope osteoporosis/osteopenia SMQ signal. This discordance indicates that absence of a fracture signal should not imply skeletal safety. These findings provide evidence-based guidance for individualized PPI prescribing in elderly patients, with careful benefit-risk assessment for those requiring extended-duration PPI regimens.

## 1 Introduction

Proton pump inhibitors (PPIs) are the first-line pharmacological therapy for acid-related disorders, including gastroesophageal reflux disease (GERD), peptic ulcer disease, and eosinophilic esophagitis (National Institute for Health and Care Excellence (NICE), 2019; Kantor et al., 2015). As one of the most widely prescribed medications globally, PPIs are highly prevalent in adult populations. A recent systematic review of global trends and practices estimated that approximately 23.4% of the adult source population worldwide were PPI users, and that adults aged ≥65 years accounted for 37.1% of all PPI users (Shanika et al., 2023).

The high prevalence of prolonged PPI use, particularly among older adults, likely reflects both evidence-based clinical indications and potentially inappropriate continuation of therapy. For chronic GERD, the most common indication in elderly populations, maintenance therapy is often necessary to prevent symptom recurrence, avoid mucosal complications, and preserve quality of life (Ahmad et al., 2025). Additionally, PPIs are the cornerstone of gastroprotection for patients at high risk of mucosal injury, including older adults receiving extended-duration non-steroidal anti-inflammatory drugs (NSAIDs) (Trukhan and Filimonov, 2024) for osteoarthritis or antiplatelet drugs (e.g., P2Y12 receptor antagonists) for cardiovascular disease, both of which impair gastrointestinal mucosal defense, neovascularization and ulcer healing (Di Minno et al., 2015); Other persistent conditions, including refractory peptic ulcer, Zollinger-Ellison syndrome, and post *Helicobacter pylori* eradication management (Jensen, 2006), further contribute to the need for sustained PPI exposure in vulnerable older populations.

Moreover, long-term PPI use in older adults is not always supported by a clear ongoing indication. Recent epidemiological studies have shown that 45.9% of long-term PPI prescriptions in multimorbid older adults lacked appropriate indications (Aubert et al., 2023), and 78.7% of PPI use beyond recommended durations (Rodrigues et al., 2024). These findings suggest that prolonged PPI exposure in older adults is driven not only by legitimate clinical need but also by potentially inappropriate continuation, underscoring the public-health relevance of evaluating long-term skeletal safety in this population.

Accumulating evidence has linked prolonged PPI use to a spectrum of adverse effects, including chronic kidney disease, hypomagnesemia, cognitive impairment, and skeletal fracture (Chaudhry et al., 2025). With respect to bone health, the proposed biological mechanisms centers on PPI-induced inhibition of gastric acid secretion, which may impair intestinal calcium absorption, disrupt physiological bone turnover, and ultimately increase fracture susceptibility (Philippoteaux et al., 2024). This risk is of particular clinical concern in older adults, who already have a high baseline risk of osteoporosis and fragility fractures. However, epidemiological evidence linking PPI use to fracture remains highly inconsistent: multiple observational studies and meta-analyses have reported an increased risk of hip, vertebral, and non-vertebral fractures with sustained PPI exposure (Zhou et al., 2016; Poly et al., 2019), while other rigorously designed studies have found no significant association after comprehensive adjustment for key confounding factors (Moayyedi et al., 2019; Harding et al., 2018). These disparate findings highlight two critical unresolved issues in the field: residual confounding in observational analyses, and the widespread practice of treating all PPIs as a uniform therapeutic class, overlooking potential heterogeneity in their skeletal safety profiles.

Notably, existing research has largely failed to systematically evaluate differential fracture risk across individual PPI agents. This issue is clinically relevant because frequent inter-PPI switching in routine practice may introduce exposure misclassification and obscure molecule-specific associations (Liu et al., 2020). Although limited clinical and observational evidence has suggested possible heterogeneity among PPIs, the available data remain fragmented. A small single-center prospective study reported that esomeprazole was associated with a greater decline in femoral neck bone mineral density T-scores than lansoprazole or pantoprazole (Ozdil et al., 2013), while another small prospective study reported molecule-specific associations between PPI use and bone mineral density (Bahtiri et al., 2016). In addition, a large prospective cohort study of older Australian women also observed different association patterns between individual PPIs and osteoporosis medication initiation or fracture outcomes (Van der Hoorn et al., 2015). While several prior meta-analyses have included subgroup analyses of individual PPIs (Van der Hoorn et al., 2015; Torvinen-Kiiskinen et al., 2018; Reyes et al., 2013; Cea Soriano et al., 2014), no study to date has carried out a comprehensive quantitative synthesis of head-to-head comparative fracture risk across all commonly prescribed PPI agents, nor has any study validated these findings using large-scale real-world pharmacovigilance data, especially in the high-risk elderly population. This critical evidence gap leaves clinicians without clear, evidence-based guidance for PPI agent selection to minimize fracture risk in older patients requiring ongoing acid suppression (Cai et al., 2015).

To fill this gap, we conducted a complementary dual-method study combining a network meta-analysis to compare fracture risks across individual PPIs, and a pharmacovigilance analysis of the US FDA Adverse Event Reporting System database to validate our findings. We hypothesized that individual PPIs have heterogeneous fracture risk profiles in elderly adults, and our study aims to provide robust, clinically actionable evidence to guide personalized PPI prescribing in this high-risk population.

## 2 Materials and methods

**FIGURE 1:**
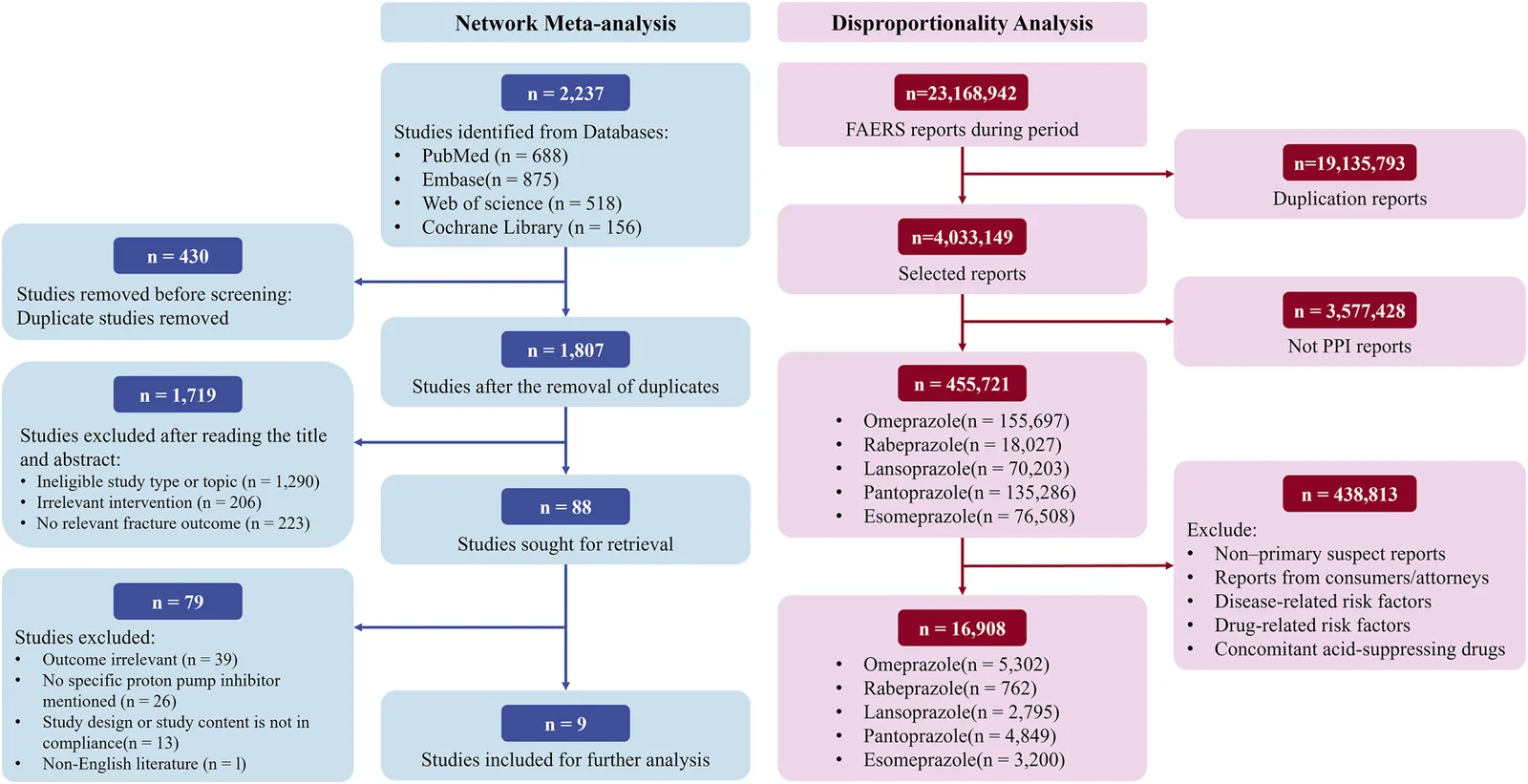
Flow diagram of the study design.

This study adopted a hybrid methodological approach integrating network meta-analysis (NMA) with disproportionality analysis based on the US FDA Adverse Event Reporting System (FAERS) database. Real-world pharmacovigilance evidence was used to complement the findings of NMA on PPI-associated fracture risk (Figure 1). Age was not used as an eligibility criterion for study selection in the network meta-analysis. However, because most eligible studies predominantly enrolled older adults or reported age-adjusted estimates, and because fracture risk is clinically most relevant in older populations, the subsequent FAERS analyses were restricted to individuals aged ≥65 years to enhance clinical comparability between the two evidence sources.

### 2.1 Network meta-analysis

#### 2.1.1 Data sources and literature search

This systematic review and NMA was performed and reported in accordance with the Preferred Reporting Items for Systematic Reviews and Meta-Analyses (PRISMA) statement and its extension for network meta-analyses (PRISMA-NMA) (Hutton et al., 2015). The study protocol was prospectively registered in the International Prospective Register of Systematic Reviews (PROSPERO, CRD420251056388). A comprehensive systematic literature search was performed across four electronic databases, including PubMed, Embase, Web of Science, and the Cochrane Library, with the search timeframe ending on 20 October 2025. The search strategy combined subject terms and free words related to proton pump inhibitors (including omeprazole, lansoprazole, pantoprazole, rabeprazole, esomeprazole, dexlansoprazole) and fracture outcomes, etc., and the detailed search strategy is provided in Supplementary Table S1.

#### 2.1.2 Study selection criteria

Inclusion criteria were set as follows: (1) Observational studies (cohort and case-control studies) that compared the fracture risk of PPI therapy with non-PPI use or placebo; (2) Studies that clearly specified the individual PPIs agents evaluated (omeprazole, lansoprazole, pantoprazole, rabeprazole, esomeprazole, or dexlansoprazole); (3) Studies that reported detailed quantitative data on fracture outcomes, including any-site fractures and/or specific fractures.

Exclusion criteria included: (1) Duplicate publications; (2) Non-original studies such as case reports, reviews, meta-analyses, letters, as well as animal or *in vitro* cell experiments; (3) Articles published in non-English languages; (4) Studies with insufficient or unavailable data for outcome extraction and analysis.

#### 2.1.3 Data extraction

Two independent reviewers (Z.H.W., and C.H.L.) executed study eligibility screening in two consecutive stages: title and abstract filtering, followed by full-text selection. Inter-rater reliability was assessed using Cohen’s κ coefficient. Any discrepancies arising during the screening process were resolved through consensus with a third independent reviewer (H.L.P.).

A standardized data extraction form, pretested on four eligible studies, was used to extract study characteristics by one reviewer (Z.H.W.), with the extracted data cross-checked and verified by a second reviewer (Z.Y.T.). The obtained study characteristics included general study information (author, publication year, journal, DOI, study design), participant demographic data, and treatment-related details (intervention and control groups, PPI type, dosage, follow-up duration). Extracted exposure-related variables included PPI type, dose, follow-up duration, and exposure definition when available. However, dose, cumulative exposure, and adherence of each PPI were not consistently reported across studies; therefore, these variables were not included in quantitative comparative analyses.

Outcome-related data for safety assessment were independently gained by two reviewers (Z.H.W., and Y.H.Y.). Prior to extraction, a third researcher (H.L.P.) de-identified all PDF files of included studies, removing information on authors, publication journals, and primary results. The two extractors performed data extraction solely based on the de-identified documents, remaining blinded to study identifiers and key findings. Extracted data included the absolute number of hip fractures and/or any-site fractures in the PPI-exposed and comparator groups. For studies with incomplete or unclear data, attempts were made to contact the corresponding authors and relevant pharmaceutical companies to obtain complete and updated original data.

#### 2.1.4 Study quality assessment

Two independent reviewers (Z.H.W., and H.L.P.) assessed the risk of bias of all included observational studies using the Newcastle-Ottawa Scale (NOS; Wells et al., 2013), The NOS evaluates study quality across three key domains: participant selection, group comparability, and outcome/exposure assessment, with a total score ranging from 0 to 9 points. A higher NOS score indicated a lower risk of bias and higher methodological quality of the study.

#### 2.1.5 Data synthesis and statistical analysis

The primary outcomes of this NMA were the risk of any-site fracture and hip fracture. First, conventional pairwise meta-analyses were performed to compare the fracture risk of each individual PPI with other exposures (other PPI types or non-PPI use), with results presented as odds ratios (OR) and their corresponding 95% confidence intervals (CI). Subsequently, frequentist random-effects NMA was done using a graph theoretical approach, which integrated the entire evidence network to estimate both direct and indirect comparative evidence between interventions, thus providing the most comprehensive assessment of relative fracture risk across PPI agents. Multi-arm studies contributing more than two PPI or comparator arms were entered as multi-arm designs rather than as independent pairwise comparisons. The frequentist graph-theoretical model accounts for within-study correlations among comparisons sharing a common comparator, thereby avoiding artificial narrowing of confidence intervals.

The transitivity assumption was assessed by examining the distribution of potential effect modifiers across treatment comparisons, including participant characteristics (age and sex), duration of follow-up, fracture outcome definitions, and adjustment for major fracture-related confounders. Statistical heterogeneity across included studies was assessed using the Higgins I^2^ statistic and the Cochran’s Q test. Significant heterogeneity was defined as I^2^ > 50% and a two-sided p < 0.05. Given the potential for high heterogeneity in observational studies, all meta-analyses were conducted using random-effects models. Inconsistency between direct and indirect evidence in the network was evaluated using the separating indirect from direct evidence (SIDE) method with back-calculation; a two-sided p < 0.05 indicated statistically significant inconsistency. Network plots were generated to visualize the evidence network, where nodes represented different interventions (PPI types/non-use), node size was proportional to the total sample size of the corresponding intervention, connecting lines indicated direct pairwise comparisons between two interventions, and line width reflected the number of studies reporting that specific comparison.

The surface under the cumulative ranking curve (SUCRA) and p-scores were used to rank the relative fracture risk of each PPI agent. Both SUCRA and p-score values range from 0 to 1, with higher values indicating a greater probability of an agent being associated with a higher fracture risk (worse outcome), and lower values indicating a higher probability of a lower fracture risk (safer outcome). Publication bias across included studies was assessed using funnel plots and Egger’s linear regression test.

Sensitivity analyses were performed to evaluate the robustness of the pooled effect estimates and explore potential sources of heterogeneity. First, a leave-one-out approach was applied, in which each study was sequentially excluded and the NMA was re-run to examine the influence of individual studies on the overall pooled results. Second, because Adams et al. (2014), Labenz et al. (2020) included mixed-age populations rather than exclusively older adults, an additional age-consistency sensitivity analysis was performed by excluding these two studies simultaneously.

### 2.2 Disproportionality analysis

#### 2.2.1 Data sources

Disproportionality analysis was undertaken using adverse event (AE) data obtained from the FAERS database, with the study timeframe covering the first quarter of 2004 to the second quarter of 2025 (data retrieved from the official FDA website). To ensure data quality, duplicate AE reports in the FAERS database were removed, and only the most recent report for each patient with complete age information was retained. The pharmacovigilance analysis was restricted to patients aged ≥65 years. PPI exposure was defined as the use of one of five commonly prescribed PPIs: omeprazole, lansoprazole, pantoprazole, rabeprazole, and esomeprazole. Extracted exposure-related variables included PPI type, dose, follow-up duration, cumulative exposure, adherence, and exposure definition when available. However, dose, follow-up duration, cumulative exposure, and adherence information were missing in more than 50% of FAERS reports and were therefore not included in the analyses.

#### 2.2.2 Definition of adverse events

Adverse events were coded and classified using the Medical Dictionary for Regulatory Activities (MedDRA). Fracture outcomes were defined based on a curated list of MedDRA preferred terms (PTs), including lumbar vertebral fracture, spinal compression fracture, hip fracture, and other relevant PTs (Supplementary Table S2). Hip fracture was additionally analyzed as an independent secondary outcome to investigate site-specific fracture risk. Moreover, osteoporosis-related outcomes were examined using the Standardised MedDRA Query (SMQ) for “Osteoporosis/Osteopenia” under both broad and narrow definitions, with the corresponding PTs detailed in Supplementary Table S2. Non-cases reports were defined as those without any fracture- or osteoporosis-related MedDRA PTs; multiple qualifying PTs within a single AE report were counted only once to avoid overestimation.

#### 2.2.3 Data deduplication and cleaning

Given the self-reported nature of the FAERS database, duplicate, withdrawn, or deleted AE reports are common. This study strictly followed the official data cleaning and deduplication guidelines provided by the FDA to ensure data integrity and accuracy. The deduplication process was performed in two steps: first, the FDA-recommended method was applied using the PRIMARYID, CASEID, and FDA_DT fields from the FAERS DEMO table. Records were sorted by CASEID, followed by FDA_DT and PRIMARYID; for records with the same CASEIDs, only the one with the most recent FDA_DT was retained, and if CASEID and FDA_DT were identical, the record with the highest PRIMARYID was kept. Second, for data from the first quarter of 2019 onwards, additional deletions were performed by removing reports listed in the official FDA deleted report list for each quarter based on CASEID.

#### 2.2.4 Statistical analysis

Descriptive statistics analysis were first performed to summarize the baseline characteristics of the study population, including patient demographics, reporting region, reporter type, and clinical outcomes.

Disproportionality analyses were estimated to quantify the association between each individual PPI and fracture-related adverse events using three validated and widely used methods: reporting odds ratio (ROR) (van Puijenbroek et al., 2002), proportional reporting ratio (PRR) (Setyawan et al., 2021), and information component (IC) (Almenoff et al., 2003) based on the Bayesian confidence propagation neural network (BCPNN) (Villa-Zapata et al., 2022). The lower bounds of the 95%CIs for ROR (ROR025) and IC (IC025) were calculated for each PPI; a statistical shrinkage transformation was applied to IC025 to ensure conservative and robust results (Norén et al., 2013; Zhai et al., 2019). A significant positive signal for PPI-associated fracture risk was defined as ROR025 > 1, PRR025 > 1, and IC025 > 0, with a minimum of 4 case reports for the specific AE-PPI combination (Noguchi et al., 2019; Yu and Liao, 2022; Setyawan et al., 2021), in accordance with standard pharmacovigilance guidelines (van Puijenbroek et al., 2002). The detailed calculation formulas for the ROR, PRR and IC are provided in Supplementary Table S3.

To minimize potential confounding by concomitant medications and indication bias and enhance the credibility of the analysis, five hierarchical analytical models were constructed with progressively stricter case selection criteria. The standard included restricting reports to those where the PPI was the primary suspect drug, excluding patients with major comorbidities or concomitant medications known to be associated with fracture risk, and removing cases involving other acid-suppressing agents (detailed in Supplementary Table S4). Model 5 was selected as the primary model because it provided the highest specificity for attributing the adverse event to the individual PPI by restricting to primary suspect reports and excluding major competing explanations. However, this increased specificity came at the cost of lower case counts. Therefore, results from Models 1 to 4 were used as sensitivity analyses to assess the consistency and robustness of the risk signals under different assumptions. Additionally, prespecified exploratory subgroup analyses were conducted by age category (65–74 years, 75–84 years, ≥85 years) and sex to explore potential heterogeneity in fracture risk signals across different subgroups of elderly patients.

All statistical analyses were performed using R software (version 4.4.1) and independently reviewed and verified by L.Y.Z.

## 3 Results

### 3.1 Network meta-analysis

#### 3.1.1 Study selection, baseline characteristics, and methodological quality

The initial systematic literature search retrieved 2237 relevant records across four electronic databases. After title and abstract screening, 88 articles underwent full-text assessment, of which 79 were excluded for not meeting the prespecified inclusion and exclusion criteria. Inter-rater reliability was excellent for both title and abstract screening (Cohen’s κ = 0.95, 95% CI 0.91–0.98) and full-text screening (Cohen’s κ = 0.93, 95% CI 0.81–1.00). Finally, 9 eligible observational studies (6 case-control studies and 3 cohort studies) enrolling a total of 257,445 elderly participants were included in the quantitative NMA, covering five commonly prescribed PPIs: omeprazole, lansoprazole, pantoprazole, rabeprazole, and esomeprazole. No studies on dexlansoprazole met the inclusion criteria, so this agent was not included in subsequent analyses (Figure 1). The evidence base should be interpreted with caution because only nine observational studies were eligible for and Lee et al. (2013) alone contributed approximately 48% of the total pooled sample size. The detailed baseline characteristics of all included studies are summarized in Supplementary Table S5. Examination of the extracted study characteristics demonstrated that the included studies were broadly comparable with respect to participant characteristics, follow-up duration, fracture outcome definitions, and adjustment for major fracture-related confounders, supporting the plausibility of the transitivity assumption.

Methodological quality and risk of bias of the included studies were appraised using the Newcastle-Ottawa Scale (NOS) (Supplementary Table S6). The overall risk of bias was low across enrolled studies: the average NOS score was 7.5 for cohort studies and 7.33 for case-control studies, with 8 of 9 (88.9%) studies rated as high quality (NOS score ≥7/9).

Heterogeneity and inconsistency confirmed the reliability of the NMA model. The results showed significant moderate-to-high heterogeneity across the evidence network: global I^2^ = 62.7%, p < 0.0001 for any-site fracture; global I^2^ = 61.9%, p = 0.0002 for hip fracture. No significant inconsistency was detected in the evidence network for either the primary outcome of any-site fracture (Supplementary Figure S1) or the secondary outcome of hip fracture (Supplementary Figure S2). Funnel plots for both endpoints were visually symmetric, and Egger’s test revealed no statistically significant evidence of publication bias (any-site fracture: p = 0.4976; hip fracture: p = 0.2532; Supplementary Figure S3).

#### 3.1.2 Pairwise network comparisons and intervention ranking

**FIGURE 2:**
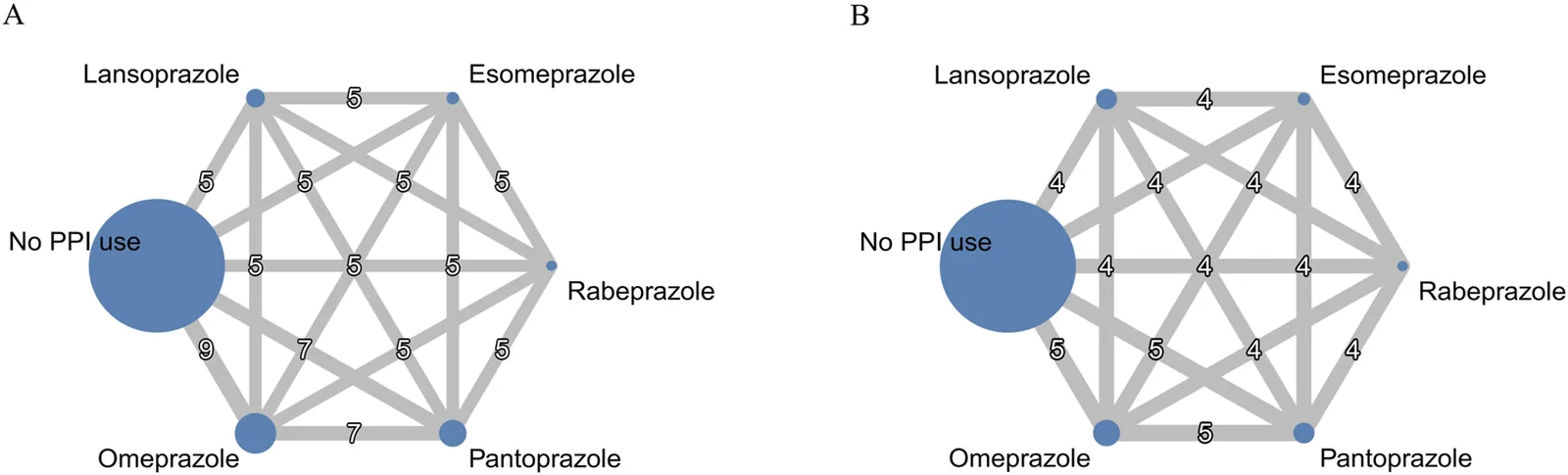
Network plots of proton pump inhibitors and fracture risk: **(A)** fracture at any site; **(B)** hip fracture. Circular nodes denote treatments, with each node’s size proportional to the total number of patients assigned to that treatment. Lines signify direct comparisons; the width of each line corresponds to the number of studies (indicated beside the line) examining the respective comparison. “No PPI use” aggregates control participants from all studies and therefore is not directly comparable to single-molecule nodes.

The network plot of all included comparisons is shown in Figure 2. Pairwise network meta-analysis was carried out to quantify the relative fracture risk of each PPI, with results presented as odds ratios (OR) with 95% confidence intervals (CI).

**FIGURE 3:**
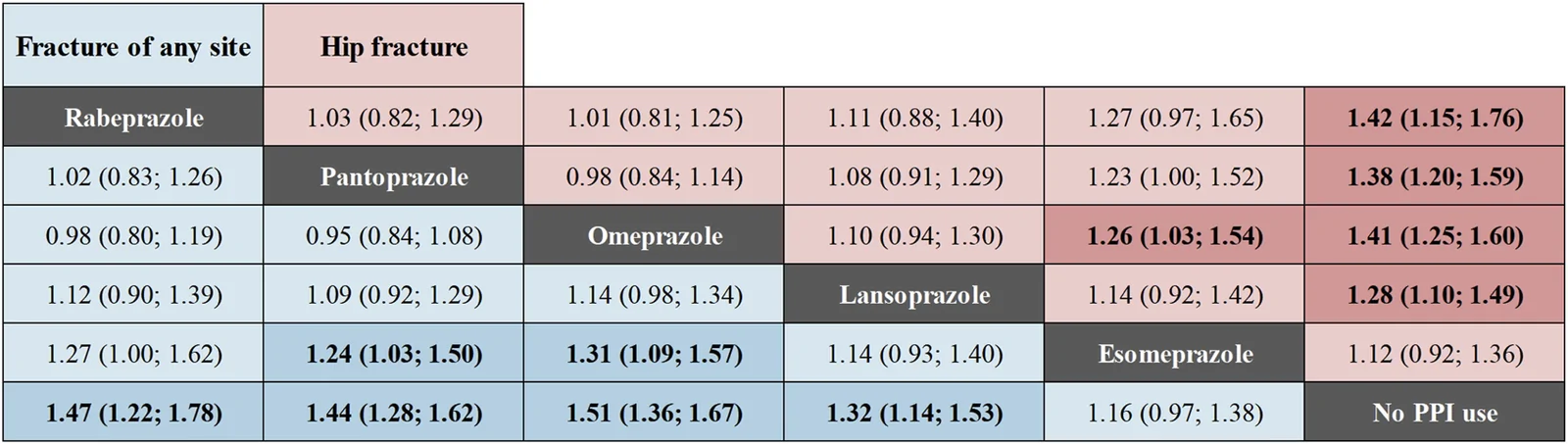
League Charts of the network meta-analysis. Each cell shows the effect size, presented as OR with 95% CIs. Results of fracture at any site are reported in the lower left part of the table (column versus row) and results of hip fracture are reported in the upper right part of the table (row versus column). An OR greater than 1 indicates a higher risk in the comparison, whereas an OR less than 1 indicates a lower risk. For example, the cell at the column “Omeprazole” and row “No PPI use” shows an OR of 1.51 (95%CI 1.36–1.67), indicating a 51% increased risk of any-site fracture in omeprazole users compared with non-PPI users. Bold ORs indicate statistically significant results. OR = Odds Ratio.

For the primary outcome of any-site fracture, compared with non-PPI users, exposure to omeprazole (OR 1.51, 95%CI: 1.36–1.67), rabeprazole (OR 1.47, 95%CI 1.22–1.78), pantoprazole (OR 1.44, 95% CI 1.28–1.62), and lansoprazole (OR 1.32, 95% CI 1.14–1.53) was associated with significantly increased risk of any-site fracture. In contrast, esomeprazole exposure showed no significant association with elevated any-site fracture risk (OR 1.16, 95%CI 0.97–1.38). In pairwise comparisons to esomeprazole, only omeprazole (OR 1.31, 95% CI 1.09–1.57) and pantoprazole (OR 1.24, 95% CI 1.03–1.50) had a significantly higher risk of any-site fracture. All other pairwise comparisons between individual PPIs did not reach statistical significance (Figure 3). These findings were fully consistent with the results of conventional pairwise meta-analyses (Supplementary Figure S4).

For the secondary outcome of hip fracture, compared with non-PPI users, omeprazole (OR 1.41, 95%CI 1.25–1.60), rabeprazole (OR 1.42, 95% CI 1.15–1.76), pantoprazole (OR 1.38, 95% CI 1.20–1.59), and lansoprazole (OR 1.28, 95%CI 1.10–1.49) were associated with a significantly increased hip fracture risk. No significant association was observed between esomeprazole exposure and hip fracture risk (OR 1.12, 95%CI 0.92–1.36). Pairwise comparisons showed that only omeprazole (OR 1.26, 95% CI 1.03–1.54) had a significantly higher hip fracture risk than esomeprazole. All other pairwise comparisons between individual PPIs did not reach statistical significance (Figure 3). These results were also consistent with conventional pairwise meta-analyses (Supplementary Figure S5).

**FIGURE 4:**
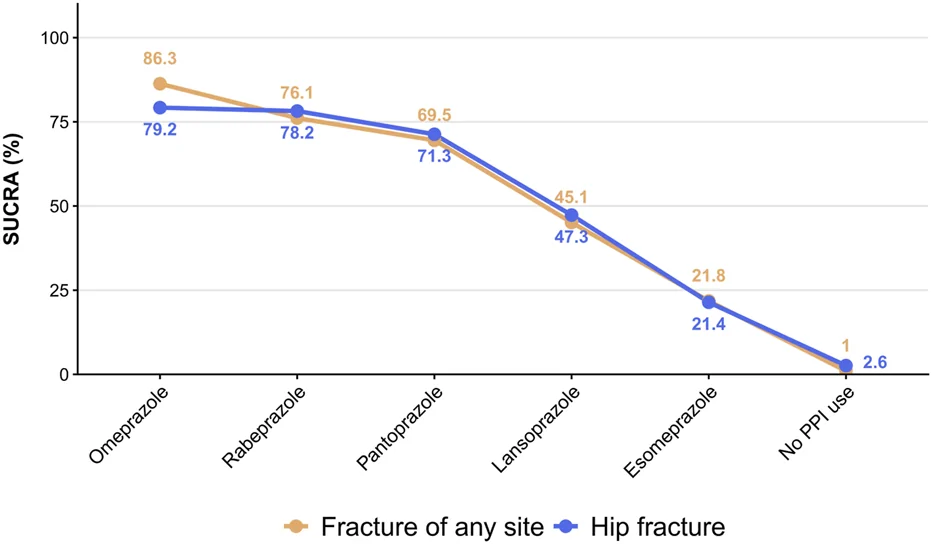
SUCRA evaluations of Specific Proton Pump Inhibitors and Fracture Risk. SUCRA, surface under the cumulative ranking curve.

Cumulative ranking of each of the five assessed PPIs by fracture risk is shown in Figure 4 and Supplementary Table S7. For any-site fracture, the ranking order from highest to lowest risk based on the surface under the cumulative ranking curve (SUCRA) was: omeprazole (SUCRA 86.3, mean rank 1.7) > rabeprazole (SUCRA 76.1, mean rank 2.2) > pantoprazole (SUCRA 69.5, mean rank 2.5) > lansoprazole (SUCRA 45.1, mean rank 3.7) > esomeprazole (SUCRA 21.8, mean rank 4.9) > no PPI use (SUCRA 1.0, mean rank 5.9). For hip fracture, the ranking order was consistent: omeprazole (SUCRA 79.2, mean rank 2.0) > rabeprazole (SUCRA 78.2, mean rank 2.1) > pantoprazole (SUCRA 71.3, mean rank 2.4) > lansoprazole (SUCRA 47.3, mean rank 3.6) > esomeprazole (SUCRA 21.4, mean rank 4.9) > non-PPI use (SUCRA 2.6, mean rank 5.9). p-scores were fully concordant with SUCRA values for both outcomes, confirming that omeprazole was associated with the highest hip fracture risk, whereas esomeprazole had the lowest relative fracture risk. Although the SUCRA analysis suggested a relative ranking of fracture risk among PPIs, most pairwise comparisons between individual PPIs were not statistically significant, and the estimated associations were generally modest. It should be particularly noted that the SUCRA ranking of rabeprazole is subject to considerable uncertainty, mainly due to the small number of included studies and low precision of its effect estimate.

#### 3.1.3 Sensitivity analysis

Leave-one-out sensitivity analyses were undertaken to evaluate the robustness of the primary NMA findings. The results confirmed that the pooled effect estimates and risk ranking of the assessed PPIs were highly robust for both any-site and hip fractures. Specifically, SUCRA values and ranking positions of omeprazole, rabeprazole, pantoprazole, and lansoprazole exhibited only minor, clinically non-significant fluctuations with sequential exclusion of individual studies, consistent with the non-significant pairwise differences between these four formulations in the primary analysis. Crucially, the risk ranking of esomeprazole remained stable across all sensitivity analyses, with its fracture risk consistently ranking the lowest among all assessed PPIs (Supplementary Table S8).

Because Lee et al. (2013) contributed approximately 48% of the pooled sample size, we further performed a dedicated leave-one-out analysis excluding this study. The resulting league table (Supplementary Figure S6) demonstrated that the pairwise treatment estimates remained largely consistent with the primary analysis. Although slight numerical changes in odds ratios and SUCRA values were observed, the overall treatment hierarchy was preserved, with omeprazole remaining the highest-ranked PPI for fracture risk. These findings suggest that the overall conclusions of the network meta-analysis were not driven by this single large study.

In addition to the leave-one-out analyses, we performed an age-consistency sensitivity analysis by simultaneously excluding Adams et al., 2014 and Labenz et al., 2020. The results were broadly consistent with the primary analysis. For any-site fracture, the direction of the associations for individual PPIs remained unchanged, and the relative ranking pattern was not materially altered. Detailed estimates from this sensitivity analysis are provided in Supplementary Table S8.

### 3.2 Disproportionality analysis of the FAERS database

#### 3.2.1 Baseline characteristics of included adverse event reports

A total of 16,908 PPI-related fracture and osteoporosis adverse events (AEs) were identified from the FAERS database in elderly patients aged ≥65 years. Among these, 5302 reports were associated with omeprazole, 762 with rabeprazole, 2795 with lansoprazole, 4849 with pantoprazole and 3200 with esomeprazole. The detailed clinical and demographic characteristics of the study population are summarized in Table 1.

**TABLE 1:** Characteristics of the patients who developed fractures after PPIs exposure from FAERS database.

Characteristic	Omeprazole	Rabeprazole	Lansoprazole	Pantoprazole	Esomeprazole
	N = 5302	N = 762	N = 2795	N = 4849	N = 3200
Sex					
Female (%)	2807 (52.94)	449 (58.92)	1560 (55.81)	2545 (52.49)	1728 (54.00)
Male (%)	2363 (44.57)	304 (39.90)	1162 (41.57)	2268 (46.77)	1382 (43.19)
Missing (%)	132 (2.49)	9 (1.18)	73 (2.61)	36 (0.74)	90 (2.81)
Age					
65-74 (%)	2362 (44.55)	342 (44.88)	1074 (38.43)	2166 (44.67)	1421 (44.41)
75-84 (%)	2074 (39.12)	294 (38.58)	1019 (36.46)	1742 (35.92)	1212 (37.88)
≥85 (%)	866 (16.33)	126 (16.54)	702 (25.12)	941 (19.41)	567 (17.72)
Median (Q1,Q3)	76 (70, 82)	75 (70, 82)	78 (71, 85)	76 (70, 83)	76 (70, 82)
Country					
China (%)	42 (0.79)	67 (8.79)	5 (0.18)	22 (0.45)	101 (3.16)
France (%)	619 (11.67)	199 (26.12)	911 (32.59)	1777 (36.65)	1315 (41.09)
Germany (%)	136 (2.57)	4 (0.52)	1 (0.04)	517 (10.66)	46 (1.44)
Japan (%)	174 (3.28)	203 (26.64)	514 (18.39)	0 (0.0)	405 (12.66)
Spain (%)	575 (10.84)	20 (2.62)	6 (0.21)	129 (2.66)	43 (1.34)
United Kingdom (%)	1786 (33.69)	6 (0.79)	669 (23.94)	74 (1.53)	127 (3.97)
United States (%)	789 (14.88)	66 (8.66)	235 (8.41)	647 (13.34)	480 (15.00)
Other (%)	1037 (19.56)	134 (17.59)	432 (15.46)	1625 (33.51)	654 (20.44)
Top 3 additional contributing countries	Netherlands 254 (4.79%)	Canada 44 (5.77%)	Italy 198 (7.08%)	Italy 376 (7.75%)	Switzerland 115 (3.59%)
​	Italy 172 (3.24%)	Australia 29 (3.81%)	Netherlands 86 (3.08%)	Canada 316 (6.52%)	Australia 99 (3.09%)
​	Sweden 75 (1.41%)	Italy 20 (2.62%)	Canada 47 (1.68%)	Netherlands 297 (6.12%)	Netherlands 96 (3.00%)
Reporter type					
Physician (%)	1947 (36.72)	379 (49.74)	1078 (38.57)	2202 (45.41)	1992 (62.25)
Pharmacist (%)	942 (17.77)	96 (12.60)	567 (20.29)	958 (19.76)	464 (14.50)
Health professional (%)	864 (16.30)	132 (17.32)	436 (15.60)	759 (15.65)	218 (6.81)
Other health-professional (%)	1548 (29.20)	155 (20.34)	713 (25.51)	930 (19.18)	526 (16.44)
Missing (%)	1 (0.02)	0 (0.0)	1 (0.04)	0 (0.0)	0 (0.0)

Reporter types were retained according to the reporter-type information recorded in FAERS. “Physician” and “Pharmacist” indicate reports submitted by medical doctors and pharmacists, respectively. “Health professional” denotes reports recorded as a non-specific healthcare-professional category without further occupational subclassification. “Other health-professional” refers to healthcare professionals other than physicians and pharmacists, such as nurses, dentists, physician assistants, or other clinical staff. Reports with missing reporter-type information were classified as missing.

Overall, AE reports were slightly more prevalent in female patients across all PPI groups, with the proportion of females ranging from 52.49% to 58.92%; highest in the rabeprazole group (58.92%). The majority of patients were aged 65–84 years, while the lansoprazole group had the highest proportion of patients aged ≥85 years (25.12%). For reporting regions, most reports for lansoprazole (32.59%), pantoprazole (36.65%), and esomeprazole (41.09%) originated from France; rabeprazole reports were mainly from Japan (26.64%) and France (26.12%); and 33.69% of omeprazole cases were from the United Kingdom. Physicians were the primary source of AE reports across all groups, accounting for the highest proportion in the esomeprazole group (62.25%).

#### 3.2.2 Disproportionality signal detection

**FIGURE 5:**
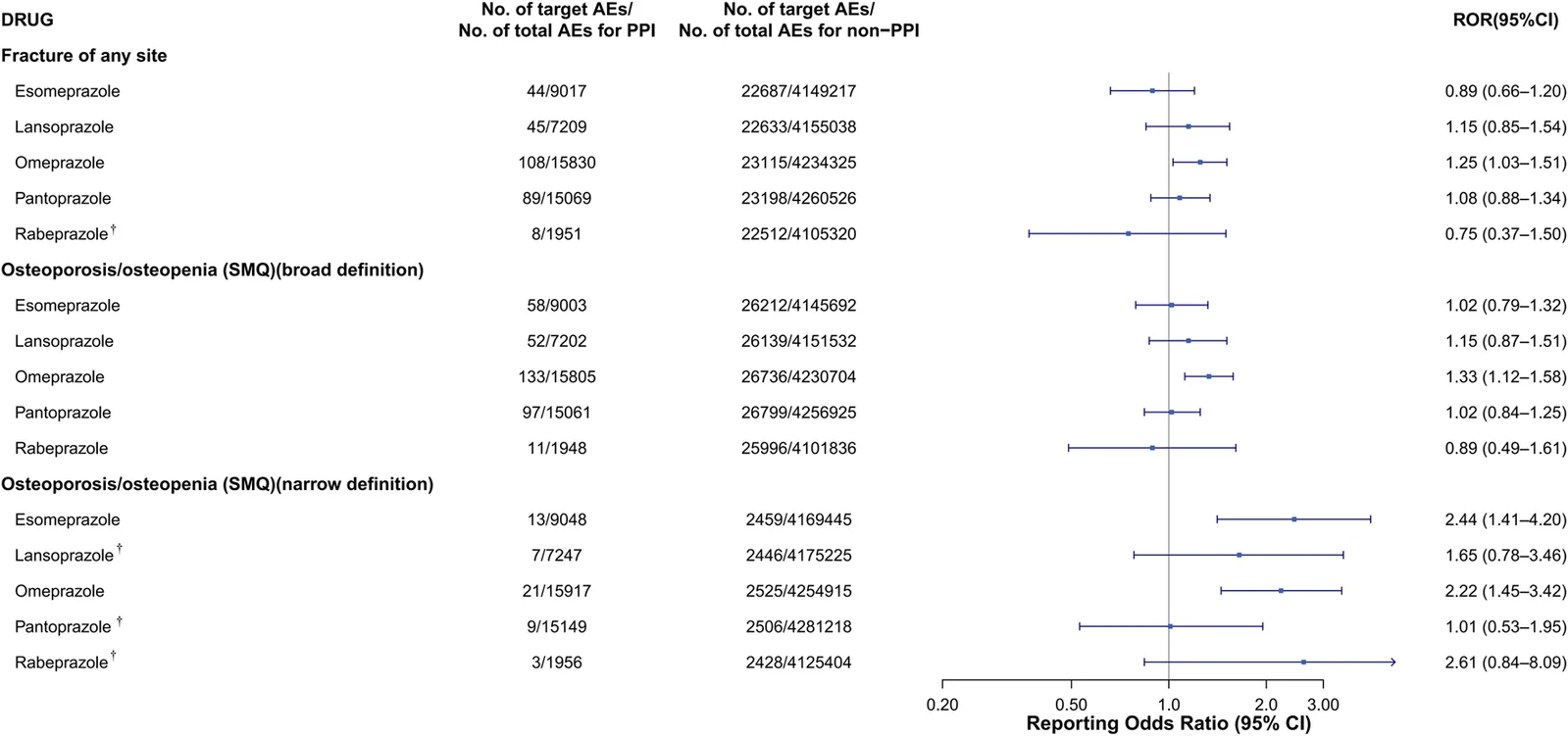
Results of disproportionality analyses for PPIs in the FAERS database. † Estimate based on fewer than 10 cases and should be interpreted as unstable. The narrow-scope SMQ included 10 preferred terms (PTs), whereas the broad-scope SMQ included more than 80 PTs; therefore, these two definitions differ substantially in specificity and sensitivity. The detailed PT lists are provided in Supplementary Table S2.

In the primary analysis using the most restrictive model (Model 5), only omeprazole demonstrated statistically significant positive risk signals for both any-site fracture (ROR 1.25, 95%CI 1.03-1.51) and the broad-scope Standardised MedDRA Query (SMQ) for Osteoporosis/Osteopenia (ROR 1.33, 95%CI 1.12-1.58). For the narrow-scope SMQ for osteoporosis/osteopenia, both esomeprazole (ROR 2.44, 95%CI 1.41-4.20) and omeprazole (ROR 2.22, 95%CI 1.45–3.42) exhibited significant positive risk signals, while no significant signals were detected for the other three PPIs (Figure 5). For rabeprazole, the any-site fracture estimate in Model 5 was based on only 8 cases; therefore, the absence of a significant signal should be regarded as data-insufficient rather than evidence of no risk. The results were consistent across the three signal detection methods (ROR, PRR and IC; Supplementary Table S9).

Sensitivity analyses using Models 1–4 with progressively less restrictive inclusion criteria were implemented to assess the robustness of the risk signals (Supplementary Figures S7–S10). Results from Models 2–4 were generally consistent with the primary analysis, with sporadic positive risk signals for the narrow-scope osteoporosis SMQ observed only for lansoprazole and rabeprazole in Models 2–3, and lansoprazole in Model 4. In contrast, Model 1 (the least restrictive criteria) yielded notably different results: esomeprazole showed positive signals across all three outcomes (any-site fracture: ROR 1.07, 95%CI 1.03–1.12; broad-scope osteoporosis SMQ: ROR 1.42, 95%CI 1.38–1.47; narrow-scope osteoporosis SMQ: ROR 2.79, 95%CI 2.61–2.97), and all PPIs exhibited significant signals for the narrow-scope osteoporosis outcome. This finding indicated that signal strength was substantially influenced by non-primary suspect drug reports and consumer-originated reports.

Across all hierarchical models, omeprazole consistently demonstrated significant risk signals across nearly all models and outcomes; pantoprazole showed no significant signal in any model; esomeprazole had no significant signals for any-site fracture or broad-scope osteoporosis outcomes in most models, but displayed stable and significant signals for the narrow-scope osteoporosis SMQ outcome. Notably, none of the PPIs showed a significant risk signal for hip fracture in the disproportionality analysis.

Predetermined subgroup analyses completed to explore population-specific heterogeneity revealed divergent risk patterns across subgroups (Supplementary Table S10). Age-stratified analysis displayed a consistent upward trend in fracture and osteoporosis-related risk signals for all formulations with advancing age (Supplementary Table S10). Sex-stratified analysis identified heterogeneous risk patterns: the fracture risk signal for omeprazole was more pronounced in female patients, whereas the osteoporosis risk signal for esomeprazole was significantly elevated in male patients (Supplementary Table S10).

## 4 Discussion

### 4.1 Principal findings

This work integrated network meta-analysis and real-world pharmacovigilance analysis to systematically compare fracture susceptibility linked to widely prescribed PPIs in elderly patients. The results suggest divergent skeletal risk profiles across the assessed agents: relative to non-exposed controls, all formulations except esomeprazole were associated with higher fracture estimates. Pairwise network comparisons further suggested that esomeprazole correlated with lower fracture risk than other agents. SUCRA scores suggested a descending ranking of fracture-related risk estimates: omeprazole, rabeprazole, pantoprazole, lansoprazole, and esomeprazole. While SUCRA scores suggested a probabilistic ranking of fracture-related risk among the assessed PPIs, these rankings should be interpreted cautiously because most head-to-head comparisons between individual PPIs were not statistically significant and the differences in estimated fracture risk were generally modest. A notable observation was that while esomeprazole presented the lowest risk for clinical fracture events, it exhibited the most robust risk signal for the narrow-scope osteoporosis SMQ outcome. Collectively, these data suggest that fracture-related associations with prolonged PPI exposure may not be uniform class-wide effect, but vary substantially by individual formulation. As such, clinical prescribing for elderly patients requiring sustained acid-suppressive therapy should be guided by the distinct skeletal risk profile of each agent.

### 4.2 Comparison with previous literature

Current findings are consistent with a general link between chronic PPI exposure and fracture susceptibility, aligning with conclusions from most prior observational investigations (Da Maia et al., 2022; Aleraij et al., 2020; Liu et al., 2019; Nassar and Richter, 2018). Beyond complementing previous class-level relationship, this analysis is, to our knowledge, the first to delineate formulation-specific risk disparities via network meta-analysis. This addresses a critical evidence gap, as prior research has often treated PPIs as a single homogeneous drug class (Liu et al., 2019; Ngamruengphong et al., 2011; Poly et al., 2019). Notably, both the network meta-analysis and FAERS database assessment consistently highlight a robust association between omeprazole use and elevated fracture risk—a conclusion supported by multiple independent lines of data (Cea Soriano et al., 2014; Torvinen-Kiiskinen et al., 2018). Although a small number of investigations report conflicting results (Gingold-Belfer et al., 2023), this inconsistency may stem from heterogeneity in study population characteristics, follow-up periods, or variable adjustment for key confounders including falls and comorbid conditions. Collectively, the cumulative evidence base is consistent with the observations from this study, suggesting that omeprazole may be associated with a comparatively higher fracture-related signal among commonly prescribed PPIs.

Furthermore, our analysis identified esomeprazole as the formulation with the lowest fracture likelihood, with its associated risk elevation not reaching statistical significance. However, esomeprazole exhibited a strong signal for the osteoporosis SMQ endpoint. Though preliminary, this observation mirrors prior published data: a prospective follow-up assessment of four PPIs found that esomeprazole was reported to be independently associated with a significant decline in lumbar spine and femoral neck bone mineral density (BMD) T-scores (Bahtiri et al., 2016); a longitudinal cohort of older Australian women documented that esomeprazole use correlated with a notable rise in osteoporosis pharmacotherapy utilization, yet no significant elevation in fracture incidence (Van der Hoorn et al., 2015). This unique pattern suggests that esomeprazole may be linked to differential effects on bone metabolism or density. However, this effect may not directly or acutely translate into clinical fracture events, potentially indicating a pathogenic mechanism distinct from other PPIs.

This finding should be interpreted cautiously because it may partly reflect differences between clinically observed fracture outcomes and osteoporosis/osteopenia coding patterns in spontaneous reports. The narrow-scope SMQ contains only 10 preferred terms, which improves specificity but may also make the signal more sensitive to diagnostic and coding practices. Country-specific reporting may also have contributed, as France and Japan accounted for approximately 54% of esomeprazole-related reports in our FAERS data. Therefore, this result should be regarded as a pharmacovigilance reporting signal rather than direct evidence of higher clinical osteoporosis risk. Although esomeprazole, the S-enantiomer of omeprazole, has been reported to exhibit stronger inhibition of PHOSPHO1 than other PPIs (Staines et al., 2021), this mechanism remains speculative and cannot explain the absence of a corresponding fracture signal. Future prospective studies incorporating longitudinal BMD assessment, bone turnover markers, detailed PPI exposure histories, and prospectively recorded falls are needed to clarify whether this discordance reflects biological effects, reporting bias, or both.

The increased fracture-related estimates for pantoprazole in the NMA were not accompanied by a significant FAERS signal. This discordance requires cautious interpretation because the two analyses reflect different evidence sources and analytical frameworks. The NMA summarizes comparative estimates from observational studies, whereas FAERS captures spontaneous reporting disproportionality. A weak or absent FAERS signal for pantoprazole may be influenced by under-reporting, primary-suspect coding, reporting practices, country-specific reporting patterns, or limited disproportionality within the FAERS database. Therefore, the pantoprazole findings should be interpreted as uncertain and should be validated in future studies with more detailed exposure and outcome ascertainment.

### 4.3 Potential mechanisms

PPIs may impact skeletal health via multiple divergent biological pathways, with fracture representing a multifactorial clinical endpoint of these effects. The canonical mechanistic explanation centers on impaired calcium absorption secondary to gastric acid suppression, particularly for less soluble calcium formulations like calcium carbonate (Philippoteaux et al., 2024). However, other acid-suppressive agents, including H2 receptor antagonists, are not linked to a comparable elevation in fracture susceptibility, pointing to PPI-specific pathogenic pathways (Vestergaard et al., 2006). Additionally, several meta-analyses on PPIs note that while exposed patients have an elevated fracture risk, the average shift in BMD is modest (Ngamruengphong et al., 2011; Nassar and Richter, 2018; Liu et al., 2019), suggesting contributions from pathways independent of bone mass. In fact, fracture likelihood is driven not only by BMD but also by bone quality and microarchitectural integrity. For instance, *in vitro* studies suggest that PPIs can suppress the bone-specific phosphatase PHOSPHO1, whose inhibition impairs bone matrix mineralization and thereby directly compromises bone quality (Staines et al., 2021). Other contributing drivers include falls, muscle weakness, and comorbidities that impact the neuromuscular system (Nassar and Richter, 2018). PPIs may increase fall risk via induction of hypomagnesemia, which impairs muscle and nerve function (Thong et al., 2019). They may also exacerbate mild confusion in patients with liver disease (Labenz et al., 2020). These effects may compromise balance and cognitive function. Falls are the primary trigger for most fragility fractures. Therefore, these agents may influence relevant pathways to different degrees. This may account for the formulation-specific risk disparities observed in this work.

### 4.4 Clinical implications

It is noteworthy that observations diverge substantially across study methodologies (Gingold-Belfer et al., 2023; Labenz et al., 2020; Moayyedi et al., 2019; Van der Hoorn et al., 2015). A large randomized controlled trial (RCT) demonstrated no statistically significant divergence between daily pantoprazole and placebo for fracture endpoints (Moayyedi et al., 2019). This difference likely arises from multiple drivers. First, RCTs mitigate confounding via random allocation, which may attenuate the measurable effect of PPIs. Second, as fractures were not a primary endpoint in this trial, the number of events over a 3-year follow-up may have been insufficient, resulting in restricted statistical power to exclude a small increase in risk. Third, this trial evaluated pantoprazole only, which limits the generalizability of its findings to other PPI agents and may not fully capture potential molecule-specific skeletal effects. This discrepancy also implies that the elevated hazard identified in observational studies may be partially shaped by unadjusted confounders. Variables such as advanced age, multimorbidity, polypharmacy, and overall health status may affect the actual incidence of fractures across cohorts, thereby confounding assessments of the agents’ independent effects (Nassar and Richter, 2018; Kwok et al., 2011).

Therefore, when interpreting the results of this study and similar observational investigations, inherent biases require careful consideration. However, this does not diminish the clinical value of observational findings. Such analyses can enroll real-world, clinically heterogeneous patient cohorts and implement extended follow-up periods, making them more skilled at detecting emerging risk signals in these high-risk clinical scenarios. In contrast, the rigid eligibility criteria of RCTs may restrict the external validity of their findings to high-risk populations encountered in routine clinical practice. Accordingly, evidence from RCTs and observational studies is inherently complementary. While RCTs provide the highest level of evidence for causal inference, care is required when extrapolating their findings to the most medically complex older patients in clinical settings. Future validation from large, rigorously designed investigations is needed to confirm these findings.

While this study offers novel insights into formulation-specific skeletal risk disparities, the impact of specific prescribing patterns including dosage, treatment duration, and sequential switching between multiple PPI agents on fracture risk has yet to be fully elucidated. Our analysis was limited by the granularity of source data, which prevented robust quantification of dose-response gradients and extended exposure effects. A prior meta-analysis indicated that longer PPI treatment duration is associated with a greater relative risk of fracture (Kwok et al., 2011). However, supporting data remain inconsistent; for instance, one investigation found an elevated risk of hip fracture following short-term use, but the association was not significant with chronic exposure (Cea Soriano et al., 2014). This divergence may be explained by “user-survivor” confounding, as patients intolerant of early adverse effects switch therapies, leaving a selectively tolerant long-term user cohort that may mask the underlying risk signal. Markedly, exposure to multiple sequential agents may correlate with greater fracture susceptibility (Van der Hoorn et al., 2015). Inter-PPI switching may occur because of formulary changes, cost, availability, tolerability, or clinician preference, and that this issue may partly explain inconsistencies in previous studies. To clarify the risk of individual agents, this study excluded patients exposed to multiple PPIs. While enhancing analytical specificity, this approach may underestimate the overall risk in routine clinical practice. Future investigations should rigorously characterize prescribing patterns of different PPIs to clarify their independent and cumulative effects on fracture risk.

The findings of this study have implications for clinical practice, but they should not be interpreted as supporting routine substitution with a specific PPI agent. For older adults requiring sustained PPI therapy, especially those with fracture or osteoporosis risk factors, treatment should follow evidence-based indications, regular reassessment of ongoing need, and use of the lowest effective dose. Because inappropriate PPI prescribing often reflects continuation after short-term indications have resolved, systematic deprescribing review, step-down regimens, and on-demand therapy should be considered where clinically appropriate (Freedberg et al., 2017). The observation that calcitriol therapy showed no clinical benefit in esomeprazole users (Huang et al., 2014) further highlights the importance of preventing avoidable skeletal harm through appropriate PPI prescribing. For long-term users at elevated fracture risk, clinicians should also consider bone-health optimization, including adequate calcium and vitamin D intake, osteoporosis risk assessment, and bone mineral density monitoring when indicated.

### 4.5 Limitations

This study has several limitations. First, network meta-analysis included only observational studies. Despite statistical adjustments, residual confounding from unmeasured or inconsistently reported factors (e.g., calcium/vitamin D supplementation, glucocorticoid exposure, physical activity, and individual fall risk) cannot be fully excluded. Second, the absence of data on specific PPI exposure (dosage and treatment duration) precluded evaluation of dose-response relationships and treatment-duration stratification, which may affect the precision of the effect estimates. Third, the analysis focused primarily on any-site fracture and hip fracture endpoints; thus, the findings cannot be extrapolated to other site-specific fracture outcomes. Finally, pharmacovigilance data are prone to the inherent biases of spontaneous adverse event reporting systems. The FAERS osteoporosis/osteopenia signal may also be influenced by country-level reporting patterns. Differences in osteoporosis screening, diagnostic coding, and adverse-event reporting culture may influence whether skeletal events are coded as osteoporosis/osteopenia or as fracture. Accordingly, the analysis can only suggest potential risk signals and can be used to corroborate findings from network meta-analyses. Notably, the observed fracture risk hierarchy among PPIs in this study is not based on well-designed head-to-head direct comparison evidence, and such studies remain extremely scarce. To confirm causal relationships between individual PPI formulations and validate these observed differences, more robust head-to-head prospective cohort studies and randomized controlled trials are required for definitive validation.

## 5 Conclusion

In summary, by integrating network meta-analysis and real-world pharmacovigilance data, this study elucidates differences in fracture risk among various PPIs in elderly patients. The results indicate that long-term PPI exposure in older adults is linked to elevated fracture likelihood, with substantial heterogeneity in hazard across individual agents. Esomeprazole is associated with the lowest risk of fracture, but its potential impact on bone mass and osteoporosis warrants ongoing clinical monitoring. This study highlights the clinical necessity of individualized drug selection based on the distinct risk profiles of different PPIs. It also provides an important evidence base for updating relevant clinical guidelines and initiating preventive research in high-risk patient cohorts.

## Data Availability

The original contributions presented in the study are included in the article/Supplementary Material, further inquiries can be directed to the corresponding author.
