# Supplementary material for: Differential fracture risk among proton pump inhibitors in older adults: evidence network and pharmacovigilance validation
Source: Front Pharmacol. 2026 Aug 19;17:1835602. doi: 10.3389/fphar.2026.1835602 (PMC13533994; doi:10.3389/fphar.2026.1835602)
Supplement: Supplementary file 1 [file Supplementaryfile1.docx]

Supplementary Material

# 1 Supplementary Tables

**Table S1	Search strategy**

Table S1 A	Search in PubMed

| #1 | ((((((Proton pump inhibitors[MeSH Terms]) OR (Inhibitors, Proton Pump[Title/Abstract])) OR (Proton Pump Inhibitor[Title/Abstract])) OR (Inhibitor, Proton Pump[Title/Abstract])) OR (Pump Inhibitor, Proton[Title/Abstract])) OR (PPIs[Title/Abstract])) OR (PPI[Title/Abstract]) |
| --- | --- |
| #2 | ((((((((Omeprazole[MeSH Terms]) OR (Prilosec[Title/Abstract])) OR (H 168-68[Title/Abstract])) OR (H 16868[Title/Abstract])) OR (H 168 68[Title/Abstract])) OR (Omeprazole Sodium[Title/Abstract])) OR (Sodium, Omeprazole[Title/Abstract])) OR (Omeprazole Magnesium[Title/Abstract])) OR (Magnesium, Omeprazole[Title/Abstract]) |
| #3 | (((((((Esomeprazole[MeSH Terms]) OR (Nexium[Title/Abstract])) OR (Esomeprazole Sodium[Title/Abstract])) OR (Esomeprazole Magnesium[Title/Abstract])) OR (Esomeprazole Potassium[Title/Abstract])) OR (Esomeprazole Strontium[Title/Abstract])) OR (Strontium, Esomeprazole[Title/Abstract])) OR (Esomeprazole Strontium Anhydrous[Title/Abstract]) |
| #4 | ((((((((((((((((((((((((Lansoprazole[MeSH Terms])) OR (Lansoprazoles[Title/Abstract])) OR (2-(((3-Methyl-4-(2,2,2-trifluoroethoxy)-2-pyridyl)methyl)sulfinyl)benzimidazole[Title/Abstract])) OR (Lansoprazol[Title/Abstract])) OR (Prevacid[Title/Abstract])) OR (Takepron[Title/Abstract])) OR (Agopton[Title/Abstract])) OR (Bamalite[Title/Abstract])) OR (Promeco[Title/Abstract])) OR (Lanzor[Title/Abstract])) OR (Monolitum[Title/Abstract])) OR (Opiren[Title/Abstract])) OR (Pro Ulco[Title/Abstract])) OR (Ulpax[Title/Abstract])) OR (Zoton[Title/Abstract])) OR (Lansol[Title/Abstract])) OR (Lansoprazole Sodium[Title/Abstract])) OR (Sodium, Lansoprazole[Title/Abstract])) OR (Ogast[Title/Abstract])) OR (Prezal[Title/Abstract])) OR (AG 1749[Title/Abstract])) OR (AG-1749[Title/Abstract])) OR (AG1749[Title/Abstract])) OR (Ogastro[Title/Abstract]) |
| #5 | (((((((((((Pantoprazole[MeSH Terms]) OR (BY 1023[Title/Abstract])) OR (BY-1023[Title/Abstract])) OR (BY1023[Title/Abstract])) OR (SK[Title/Abstract] AND F 96022[Title/Abstract])) OR (SK[Title/Abstract] AND F-96022[Title/Abstract])) OR (SK[Title/Abstract] AND F96022[Title/Abstract])) OR (SKF-96022[Title/Abstract])) OR (SKF96022[Title/Abstract])) OR (SKF 96022[Title/Abstract])) OR (Protonix[Title/Abstract])) OR (Pantoprazole Sodium[Title/Abstract]) |
| #6 | (((((((((((((Rabeprazole[MeSH Terms]) OR (2-((4-(3-methoxypropoxy)-3-methylpyridin-2-yl)methylsulfinyl)-1H-benzimidazole[Title/Abstract])) OR (E 3810[Title/Abstract])) OR (E3810[Title/Abstract])) OR (Rabeprazole Sodium[Title/Abstract])) OR (Sodium, Rabeprazole[Title/Abstract])) OR (1H-Benzimidazole, 2-(((4-(3-methoxypropoxy)-3-methyl-2-pyridinyl)methyl)sulfinyl)-, Sodium Salt[Title/Abstract])) OR (LY-307640[Title/Abstract])) OR (LY 307640[Title/Abstract])) OR (LY307640[Title/Abstract])) OR (Pariet[Title/Abstract])) OR (Dexrabeprazole[Title/Abstract])) OR (Aciphex[Title/Abstract]) |
| #7 | ((((((((((((((((((Dexlansoprazole[MeSH Terms]) OR (R-Lansoprazole[Title/Abstract])) OR (R Lansoprazole[Title/Abstract])) OR (Lansoprazole, R-Isomer[Title/Abstract])) OR (Lansoprazole, R Isomer[Title/Abstract])) OR (R-Isomer Lansoprazole[Title/Abstract])) OR (2-((R)-((3-Methyl-4-(2,2,2-trifluoroethoxy)-2-pyridinyl)methyl)sulfinyl)-1H-benzimidazole[Title/Abstract])) OR (TAK 390MR[Title/Abstract])) OR (TAK-390MR[Title/Abstract])) OR (TAK390MR[Title/Abstract])) OR (Dexilant[Title/Abstract])) OR (TAK-390[Title/Abstract])) OR (TAK390[Title/Abstract])) OR (TAK 390[Title/Abstract])) OR (T-168390[Title/Abstract])) OR (T168390[Title/Abstract])) OR (T 168390[Title/Abstract])) OR (Dexlansoprazole Sesquihydrate[Title/Abstract]) |
| #8 | acid-suppressive drug[Title/Abstract] |
| #9 | #1 OR #2 OR #3 OR #4 OR #5 OR #6 OR #7 OR #8 |
| #10 | "fractur"[All Fields] OR "fractural"[All Fields] OR "fracture s"[All Fields] OR "fractures, bone"[MeSH Terms] OR ("fractures"[All Fields] AND "bone"[All Fields]) OR "bone fractures"[All Fields] OR "fracture"[All Fields] OR "fractured"[All Fields] OR "fractures"[All Fields] OR "fracturing"[All Fields] |
| #11 | (((((((((((((((((((((Osteoporosis[MeSH Terms]) OR (Osteoporoses[Title/Abstract])) OR (Osteoporosis, Age-Related[Title/Abstract])) OR (Osteoporosis, Age Related[Title/Abstract])) OR (Age-Related Osteoporosis[Title/Abstract])) OR (Age-Related Osteoporoses[Title/Abstract])) OR (Age Related Osteoporosis[Title/Abstract])) OR (Osteoporoses, Age-Related[Title/Abstract])) OR (Bone Loss, Age-Related[Title/Abstract])) OR (Age-Related Bone Loss[Title/Abstract])) OR (Age-Related Bone Losses[Title/Abstract])) OR (Bone Loss, Age Related[Title/Abstract])) OR (Bone Losses, Age-Related[Title/Abstract])) OR (Osteoporosis, Senile[Title/Abstract])) OR (Osteoporoses, Senile[Title/Abstract])) OR (Senile Osteoporoses[Title/Abstract])) OR (Senile Osteoporosis[Title/Abstract])) OR (Osteoporosis, Involutional[Title/Abstract])) OR (Osteoporosis, Post-Traumatic[Title/Abstract])) OR (Osteoporosis, Post Traumatic[Title/Abstract])) OR (Post-Traumatic Osteoporoses[Title/Abstract])) OR (Post-Traumatic Osteoporosis[Title/Abstract]) |
| #12 | #10 OR #11 |
| #13 | #9 AND #12 |

Table S1 B	Search in Embase

| #1 | 'proton pump inhibitor'/exp |
| --- | --- |
| #2 | 'gastric proton pump inhibitor':ti,ab,kw OR 'hydrogen potassium adenosine triphosphatase inhibitor':ti,ab,kw OR 'hydrogen potassium atpase inhibitor':ti,ab,kw OR 'proton pump inhibitors':ti,ab,kw OR 'proton pump inhibitor':ti,ab,kw OR ppis:ti,ab,kw OR ppi:ti,ab,kw |
| #3 | 'omeprazole'/exp |
| #4 | (('5 methoxy 2 [ [':ti,ab,kw AND '4 methoxy 3, 5 dimethyl 2 pyridyl':ti,ab,kw AND 'methyl] sulfinyl] benzimidazole':ti,ab,kw OR '5 methoxy 2 [ [':ti,ab,kw) AND '4 methoxy 3, 5 dimethylpyrid 2 yl':ti,ab,kw AND 'methyl] sulfinyl] benzimidazole':ti,ab,kw OR aleprozil:ti,ab,kw OR antra:ti,ab,kw OR 'antra mups':ti,ab,kw OR arapride:ti,ab,kw OR audazol:ti,ab,kw OR baromezole:ti,ab,kw OR belmazol:ti,ab,kw OR desec:ti,ab,kw OR dolintol:ti,ab,kw OR domer:ti,ab,kw OR dudencer:ti,ab,kw OR duogas:ti,ab,kw OR emeproton:ti,ab,kw OR epirazole:ti,ab,kw OR ezipol:ti,ab,kw OR gasec:ti,ab,kw OR 'gasec gastrocaps':ti,ab,kw OR gastec:ti,ab,kw OR gastop:ti,ab,kw OR gastrimut:ti,ab,kw OR gastrolac:ti,ab,kw OR gastroloc:ti,ab,kw OR glaveral:ti,ab,kw OR 'h 168 68':ti,ab,kw OR 'h 168-68':ti,ab,kw OR 'h etom':ti,ab,kw OR 'h168 68':ti,ab,kw OR hovizol:ti,ab,kw OR hyposec:ti,ab,kw OR inhibitron:ti,ab,kw OR inhipump:ti,ab,kw OR logastric:ti,ab,kw OR lomac:ti,ab,kw OR lopraz:ti,ab,kw OR losamel:ti,ab,kw OR losec:ti,ab,kw OR 'losec mups':ti,ab,kw OR losecosan:ti,ab,kw OR ludea:ti,ab,kw OR madiprazole:ti,ab,kw OR maxor:ti,ab,kw OR medoprazole:ti,ab,kw OR medral:ti,ab,kw OR meiceral:ti,ab,kw OR mepral:ti,ab,kw OR mepzol:ti,ab,kw OR mezzopram:ti,ab,kw OR miol:ti,ab,kw OR miracid:ti,ab,kw OR mopral:ti,ab,kw OR mopralpro:ti,ab,kw OR nocid:ti,ab,kw OR nuclosina:ti,ab,kw OR ocid:ti,ab,kw OR ogal:ti,ab,kw OR olexin:ti,ab,kw OR omedar:ti,ab,kw OR omelon:ti,ab,kw OR 'omep uno':ti,ab,kw OR omepral:ti,ab,kw OR omeprazen:ti,ab,kw OR omeprazol:ti,ab,kw OR 'omeprazole magnesium':ti,ab,kw OR 'omeprazole sodium':ti,ab,kw OR omeprazon:ti,ab,kw OR omepril:ti,ab,kw OR omeraz:ti,ab,kw OR omesec:ti,ab,kw OR omestad:ti,ab,kw OR omezin:ti,ab,kw OR omezol:ti,ab,kw OR omezolan:ti,ab,kw OR omezole:ti,ab,kw OR omezzol:ti,ab,kw OR omisec:ti,ab,kw OR omizac:ti,ab,kw OR omolin:ti,ab,kw OR ompranyt:ti,ab,kw OR omprazole:ti,ab,kw OR onexal:ti,ab,kw OR oprax:ti,ab,kw OR ozoken:ti,ab,kw OR parizac:ti,ab,kw OR penrazole:ti,ab,kw OR pepticum:ti,ab,kw OR peptidin:ti,ab,kw OR peptilcer:ti,ab,kw OR peptizole:ti,ab,kw OR 'pra sec':ti,ab,kw OR prazidec:ti,ab,kw OR prazole:ti,ab,kw OR prilosec:ti,ab,kw OR 'prilosec otc':ti,ab,kw OR prisolec:ti,ab,kw OR probitor:ti,ab,kw OR proceptin:ti,ab,kw OR protoloc:ti,ab,kw OR ramezol:ti,ab,kw OR reglacid:ti,ab,kw OR result:ti,ab,kw) AND drug:ti,ab,kw OR risek:ti,ab,kw OR romep:ti,ab,kw OR roweprazol:ti,ab,kw OR secrepina:ti,ab,kw OR severon:ti,ab,kw OR stomacer:ti,ab,kw OR stomec:ti,ab,kw OR stozole:ti,ab,kw OR suifac:ti,ab,kw OR ulceral:ti,ab,kw OR ulcozol:ti,ab,kw OR ulnor:ti,ab,kw OR ulsek:ti,ab,kw OR ulsen:ti,ab,kw OR ulzol:ti,ab,kw OR vulcasid:ti,ab,kw OR wonmp:ti,ab,kw OR xoprin:ti,ab,kw OR zatrol:ti,ab,kw OR zefxon:ti,ab,kw OR zenpro:ti,ab,kw OR zimor:ti,ab,kw OR zoltum:ti,ab,kw OR omeprazole:ti,ab,kw |
| #5 | 'esomeprazole'/exp |
| #6 | ((esofag:ti,ab,kw OR esomeprazol:ti,ab,kw OR 'esomeprazole magnesium':ti,ab,kw OR 'esomeprazole potassium':ti,ab,kw OR 'esomeprazole sodium':ti,ab,kw OR esoprax:ti,ab,kw OR 'h 199 18':ti,ab,kw OR 'h 199-18':ti,ab,kw OR 'h 19918':ti,ab,kw OR 'h199 18':ti,ab,kw OR h19918:ti,ab,kw OR 'hip 1601':ti,ab,kw OR hip1601:ti,ab,kw OR inexium:ti,ab,kw OR izra:ti,ab,kw) AND drug:ti,ab,kw OR nexiam:ti,ab,kw OR nexium:ti,ab,kw OR 'nexium 24hr':ti,ab,kw OR 'nexium control':ti,ab,kw OR 'nexium iv':ti,ab,kw OR 'nexium mups':ti,ab,kw OR perprazole:ti,ab,kw OR sompraz:ti,ab,kw OR ulceran:ti,ab,kw) AND esomeprazole:ti,ab,kw OR esomeprazole:ti,ab,kw |
| #7 | 'lansoprazole'/exp |
| #8 | ('2 [ [[3 methyl 4':ti,ab,kw AND '2, 2, 2 trifluoroethoxy':ti,ab,kw AND '2 pyridyl] methyl] sulfinyl] 1h benzimidazole':ti,ab,kw OR 'a 65006':ti,ab,kw OR a65006:ti,ab,kw OR 'abt 006':ti,ab,kw OR abt006:ti,ab,kw OR 'ag 1749':ti,ab,kw OR ag1749:ti,ab,kw OR agopton:ti,ab,kw OR bamalite:ti,ab,kw OR banilux:ti,ab,kw OR betalans:ti,ab,kw OR compraz:ti,ab,kw OR dakar:ti,ab,kw) AND drug:ti,ab,kw OR daxar:ti,ab,kw OR dostab:ti,ab,kw OR duomate:ti,ab,kw OR ilsatec:ti,ab,kw OR inhipraz:ti,ab,kw OR keval:ti,ab,kw OR lancid:ti,ab,kw OR lancopen:ti,ab,kw OR langaton:ti,ab,kw OR lanpra:ti,ab,kw OR lanpraz:ti,ab,kw OR lanprol:ti,ab,kw OR lanproton:ti,ab,kw OR lansazol:ti,ab,kw OR lansobene:ti,ab,kw OR lansol:ti,ab,kw OR lansone:ti,ab,kw OR lansop:ti,ab,kw OR lansopep:ti,ab,kw OR lansoprazol:ti,ab,kw OR lansox:ti,ab,kw OR lansozole:ti,ab,kw OR lanster:ti,ab,kw OR lanston:ti,ab,kw OR lanvell:ti,ab,kw OR lanximed:ti,ab,kw OR lanzo:ti,ab,kw OR 'lanzol 30':ti,ab,kw OR lanzopral:ti,ab,kw OR lanzoprazole:ti,ab,kw OR lanzor:ti,ab,kw OR lanzul:ti,ab,kw OR lapraz:ti,ab,kw OR laprazol:ti,ab,kw OR laproton:ti,ab,kw OR lasgan:ti,ab,kw OR limpidex:ti,ab,kw OR lopral:ti,ab,kw OR monolitum:ti,ab,kw OR ogast:ti,ab,kw OR ogasto:ti,ab,kw OR ogastoro:ti,ab,kw OR ogastro:ti,ab,kw OR opiren:ti,ab,kw OR pampe:ti,ab,kw OR praton:ti,ab,kw OR prevacid:ti,ab,kw OR 'prevacid 24 hr':ti,ab,kw OR 'prevacid fastab':ti,ab,kw OR 'prevacid iv':ti,ab,kw OR 'prevacid solutab':ti,ab,kw OR prezal:ti,ab,kw OR prolanz:ti,ab,kw OR prosogan:ti,ab,kw OR pysolan:ti,ab,kw OR 'sopralan 30':ti,ab,kw OR suprecid:ti,ab,kw OR takepron:ti,ab,kw OR 'takepron od':ti,ab,kw OR tanzolan:ti,ab,kw OR ulpax:ti,ab,kw OR zoton:ti,ab,kw OR 'zoton fastab':ti,ab,kw OR lansoprazole:ti,ab,kw |
| #9 | 'pantoprazole'/exp |
| #10 | (((('5 difluoromethoxy 2 [':ti,ab,kw AND '3, 4 dimethoxy 2 pyridyl':ti,ab,kw AND 'methylsulfinyl] 1h benzimidazole':ti,ab,kw OR 6:ti,ab,kw) AND difluoromethoxy:ti,ab,kw AND '2 [':ti,ab,kw AND '3, 4 dimethoxy 2 pyridinyl':ti,ab,kw AND 'methylsulfinyl] 1h benzimidazole':ti,ab,kw OR 6:ti,ab,kw) AND difluoromethoxy:ti,ab,kw AND '2 [':ti,ab,kw AND '3, 4 dimethoxypyridin 2 yl':ti,ab,kw AND 'methylsulfinyl] 1h benzimidazole':ti,ab,kw OR acidwell:ti,ab,kw OR acipan:ti,ab,kw OR alapanzol:ti,ab,kw OR anagastra:ti,ab,kw OR anesteloc:ti,ab,kw OR apton:ti,ab,kw OR 'b 8610 023':ti,ab,kw OR 'b 8610 23':ti,ab,kw OR 'b8610 023':ti,ab,kw OR 'b8610 23':ti,ab,kw OR berozol:ti,ab,kw OR brandocare:ti,ab,kw OR branzol:ti,ab,kw OR 'by 1023':ti,ab,kw OR by1023:ti,ab,kw OR citrel:ti,ab,kw OR contix:ti,ab,kw OR controloc:ti,ab,kw OR 'controloc control':ti,ab,kw OR 'dicarbocalm control':ti,ab,kw OR digespan:ti,ab,kw OR dosanloc:ti,ab,kw OR 'dz 2352a':ti,ab,kw OR dz2352a:ti,ab,kw OR 'eugastrol reflusso':ti,ab,kw OR eupantol:ti,ab,kw OR 'gastroloc hexal':ti,ab,kw OR gastrostad:ti,ab,kw OR 'gaxtron start':ti,ab,kw OR gerdin:ti,ab,kw OR 'gerdin max':ti,ab,kw OR inipant:ti,ab,kw OR inipepsia:ti,ab,kw OR inipom:ti,ab,kw OR inipomp:ti,ab,kw OR inizol:ti,ab,kw OR ippracid:ti,ab,kw OR ipraalox:ti,ab,kw OR kairol:ti,ab,kw OR 'maalox control':ti,ab,kw OR 'maalox reflusso':ti,ab,kw OR nedis:ti,ab,kw OR noacid:ti,ab,kw OR nolpacid:ti,ab,kw OR nolpanta:ti,ab,kw OR 'nolpaza control':ti,ab,kw OR normogastrol:ti,ab,kw OR ozzion:ti,ab,kw OR pacid:ti,ab,kw OR pangest:ti,ab,kw OR panprazox:ti,ab,kw OR panrazol:ti,ab,kw OR 'pantacid flux':ti,ab,kw OR pantecta:ti,ab,kw OR 'pantecta control':ti,ab,kw OR pantip:ti,ab,kw OR pantium:ti,ab,kw OR 'panto aiwa':ti,ab,kw OR 'panto aristo':ti,ab,kw OR 'panto tad':ti,ab,kw OR pantodac:ti,ab,kw OR pantodar:ti,ab,kw OR pantofir:ti,ab,kw OR pantoflux:ti,ab,kw OR pantogastrix:ti,ab,kw OR pantogen:ti,ab,kw OR pantoloc:ti,ab,kw OR 'pantoloc control':ti,ab,kw OR pantomac:ti,ab,kw OR pantomol:ti,ab,kw OR pantomyl:ti,ab,kw OR pantop:ti,ab,kw OR pantopan:ti,ab,kw OR 'pantopra q':ti,ab,kw OR pantopraz:ti,ab,kw OR 'pantopraz bio':ti,ab,kw OR 'pantoprazole sodium':ti,ab,kw OR 'pantoprazole sodium sesquihydrate':ti,ab,kw OR pantopro:ti,ab,kw OR pantoprol:ti,ab,kw OR pantorena:ti,ab,kw OR pantorex:ti,ab,kw OR pantosec:ti,ab,kw OR pantozol:ti,ab,kw OR 'pantozol control':ti,ab,kw OR pantup:ti,ab,kw OR panzilan:ti,ab,kw OR panzol:ti,ab,kw OR 'panzol pro':ti,ab,kw OR peptazol:ti,ab,kw OR pepticus:ti,ab,kw OR 'pf 05208751':ti,ab,kw OR pf05208751:ti,ab,kw OR piador:ti,ab,kw OR 'praz up':ti,ab,kw OR prazolan:ti,ab,kw) AND pantoprazole:ti,ab,kw OR pronzek:ti,ab,kw OR protium:ti,ab,kw OR protizole:ti,ab,kw OR protonix:ti,ab,kw OR 'protonix iv':ti,ab,kw OR ranloc:ti,ab,kw OR 'ranloc med':ti,ab,kw OR rifun:ti,ab,kw OR 'rifun 40':ti,ab,kw OR roxitrol:ti,ab,kw OR 'rvg 22106':ti,ab,kw OR rvg22106:ti,ab,kw OR sanopaz:ti,ab,kw OR sedipanto:ti,ab,kw OR seltraz:ti,ab,kw OR sk:ti,ab,kw) AND 'f 96022':ti,ab,kw OR 'skf 96022':ti,ab,kw OR skf96022:ti,ab,kw OR sodac:ti,ab,kw OR somac:ti,ab,kw OR 'somac control':ti,ab,kw OR tecnozol:ti,ab,kw OR tecta:ti,ab,kw OR ulcepraz:ti,ab,kw OR ulceron:ti,ab,kw OR ulcotenal:ti,ab,kw OR ulprix:ti,ab,kw OR 'way 140951':ti,ab,kw OR way140951:ti,ab,kw OR 'xoolam reflusso':ti,ab,kw OR zencopan:ti,ab,kw OR zipantola:ti,ab,kw OR 'zipantola protect':ti,ab,kw OR ziprol:ti,ab,kw OR zolepant:ti,ab,kw OR zolium:ti,ab,kw OR zoltex:ti,ab,kw OR zoprax:ti,ab,kw OR zurcal:ti,ab,kw OR zurcale:ti,ab,kw OR zurcazol:ti,ab,kw OR pantoprazole:ti,ab,kw |
| #11 | 'rabeprazole'/exp |
| #12 | (((((('2 [ [4':ti,ab,kw AND '3 methoxypropoxy':ti,ab,kw AND '3 methyl 2 pyridinyl] methylsulfinyl] 1h benzimidazole':ti,ab,kw OR '2 [ [4':ti,ab,kw) AND '3 methoxypropoxy':ti,ab,kw AND '3 methyl 2 pyridyl] methylsulfinyl] benzimidazole':ti,ab,kw OR '2 [ [4':ti,ab,kw) AND '3 methoxypropoxy':ti,ab,kw AND '3 methylpyridin 2 yl] methylsulfinyl] 1h benzimidazole':ti,ab,kw OR acilesol:ti,ab,kw OR aciphex:ti,ab,kw OR 'aciphex sprinkle':ti,ab,kw OR aircodos:ti,ab,kw OR ares:ti,ab,kw) AND drug:ti,ab,kw OR bacanero:ti,ab,kw OR berlex:ti,ab,kw OR beryx:ti,ab,kw OR dexrabeprazole:ti,ab,kw OR 'e 3810':ti,ab,kw) AND 'benzimidazole derivative':ti,ab,kw OR e3810:ti,ab,kw) AND 'benzimidazole derivative':ti,ab,kw OR fodren:ti,ab,kw OR gelbra:ti,ab,kw OR 'ly 307640':ti,ab,kw OR ly307640:ti,ab,kw OR medotis:ti,ab,kw OR noflux:ti,ab,kw OR pariet:ti,ab,kw OR pariprazole:ti,ab,kw OR 'pariprazole sodium':ti,ab,kw OR pepcia:ti,ab,kw OR praber:ti,ab,kw OR rabec:ti,ab,kw OR rabecell:ti,ab,kw OR rabeloc:ti,ab,kw OR rabeman:ti,ab,kw OR rabeprazol:ti,ab,kw OR 'rabeprazole sodium':ti,ab,kw OR raberen:ti,ab,kw OR rabex:ti,ab,kw OR rabicip:ti,ab,kw OR rablet:ti,ab,kw OR rabyprex:ti,ab,kw OR ralic:ti,ab,kw OR rapoxol:ti,ab,kw OR razole:ti,ab,kw OR relitaz:ti,ab,kw OR starab:ti,ab,kw OR stom:ti,ab,kw) AND rabeprazole:ti,ab,kw OR urabis:ti,ab,kw OR yzilan:ti,ab,kw OR zulbex:ti,ab,kw OR rabeprazole:ti,ab,kw |
| #13 | 'dexlansoprazole'/exp |
| #14 | dexilant:ti,ab,kw OR 'dexilant solutab':ti,ab,kw OR dexivant:ti,ab,kw OR gladexa:ti,ab,kw OR kapidex:ti,ab,kw OR 't 168390':ti,ab,kw OR t168390:ti,ab,kw OR 'tak 390':ti,ab,kw OR 'tak 390mr':ti,ab,kw OR tak390:ti,ab,kw OR tak390mr:ti,ab,kw OR dexlansoprazole:ti,ab,kw |
| #15 | #1 OR #2 OR #3 OR #4 OR #5 OR #6 OR #7 OR #8 OR #9 OR #10 OR #11 OR #12 OR #13 OR #14 |
| #16 | 'fracture'/exp |
| #17 | 'bone cement fracture':ti,ab,kw OR 'bone fracture':ti,ab,kw OR 'bone fractures':ti,ab,kw OR 'broken bone':ti,ab,kw OR 'broken bones':ti,ab,kw OR 'closed fracture':ti,ab,kw OR 'closed fractures':ti,ab,kw OR 'fractured bone':ti,ab,kw OR fractures:ti,ab,kw OR 'fractures, bone':ti,ab,kw OR 'fractures, closed':ti,ab,kw OR 'skeleton fracture':ti,ab,kw OR 'unstable fracture':ti,ab,kw OR fracture:ti,ab,kw |
| #18 | 'osteoporosis'/exp |
| #19 | 'decalcification, pathologic':ti,ab,kw OR 'endocrine osteoporosis':ti,ab,kw OR 'osteoporotic decalcification':ti,ab,kw OR 'pathologic decalcification':ti,ab,kw OR 'osteoporosis':ti,ab,kw |
| #20 | #16 OR #17 OR #18 OR #19 |
| #21 | #15 AND #20 |
| #22 | 'cohort analysis'/exp |
| #23 | 'analysis, cohort':ti,ab,kw OR 'cohort fertility':ti,ab,kw OR 'cohort life cycle':ti,ab,kw OR 'cohort studies':ti,ab,kw OR 'cohort study':ti,ab,kw OR 'fertility, cohort':ti,ab,kw OR 'cohort analysis':ti,ab,kw |
| #24 | 'case control study'/exp |
| #25 | 'case-control studies':ti,ab,kw OR 'case-control study':ti,ab,kw OR 'control study, case':ti,ab,kw OR 'matched case control':ti,ab,kw OR 'matched case control studies':ti,ab,kw OR 'matched case control study':ti,ab,kw OR 'case control study':ti,ab,kw |
| #26 | 'nested case–control':ti,ab,kw |
| #27 | 'real-world study':ti,ab,kw |
| #28 | 'randomized controlled trial'/exp |
| #29 | 'controlled trial, randomized':ti,ab,kw OR 'randomised controlled study':ti,ab,kw OR 'randomised controlled trial':ti,ab,kw OR 'randomized controlled study':ti,ab,kw OR 'trial, randomized controlled':ti,ab,kw OR 'randomized controlled trial':ti,ab,kw OR rct:ti,ab,kw |
| #30 | #22 OR #23 OR #24 OR #25 OR #26 OR #27 OR #28 OR #29 |
| #31 | #21 AND #30 |

Table S1 C	Search in Web of Science

| #1 | TS=(Proton Pump Inhibitors OR Inhibitors, Proton Pump OR Proton Pump Inhibitor OR Inhibitor, Proton Pump OR Pump Inhibitor, Proton OR PPI OR PPIs OR ppi) |
| --- | --- |
| #2 | TS=(Omeprazole OR Esomeprazole OR Lansoprazole OR Pantoprazole OR Rabeprazole OR Dexlansoprazole OR acid-suppressive drug) |
| #3 | #1 OR #2 |
| #4 | TS=(Fracture OR Fractures, Bone OR Bone Fracture OR Fracture, Bone OR Bone Fractures OR Broken Bones OR Bone, Broken OR Bones, Broken OR Broken Bone OR Spiral Fractures OR Fracture, Spiral OR Fractures, Spiral OR Spiral Fracture OR Torsion Fractures OR Fractures, Torsion OR Fracture, Torsion OR Torsion Fracture) |
| #5 | TS=(Osteoporosis OR Osteoporoses OR Osteoporosis, Age-Related OR Osteoporosis, Age Related OR Age-Related Osteoporosis OR Age-Related Osteoporoses OR Age Related Osteoporosis OR Osteoporoses, Age-Related OR Bone Loss, Age-Related OR Age-Related Bone Loss OR Age-Related Bone Losses OR Bone Loss, Age Related OR Bone Losses, Age-Related OR Osteoporosis, Senile OR Osteoporoses, Senile OR Senile Osteoporoses OR Senile Osteoporosis OR Osteoporosis, Involutional OR Osteoporosis, Post-Traumatic OR Osteoporosis, Post Traumatic OR Post-Traumatic Osteoporoses OR Post-Traumatic Osteoporosis) |
| #6 | #4 OR #5 |
| #7 | #3 AND #6 |
| #8 | TS=(Cohort Studies OR Cohort Study OR Studies, Cohort OR Study, Cohort OR Studies, Concurrent OR Concurrent Study OR Study, Concurrent OR Concurrent Studies OR Closed Cohort Studies OR Cohort Studies, Closed OR Closed Cohort Study OR Cohort Study, Closed OR Study, Closed Cohort OR Studies, Closed Cohort OR Historical Cohort Studies OR Cohort Studies, Historical OR Cohort Study, Historical OR Historical Cohort Study OR Study, Historical Cohort OR Studies, Historical Cohort OR Incidence Studies OR Incidence Study OR Studies, Incidence OR Study, Incidence OR Analysis, Cohort OR Analyses, Cohort OR Cohort Analyses OR Cohort Analysis OR Birth Cohort Studies OR Birth Cohort Study OR Cohort Studies, Birth OR Cohort Study, Birth OR Studies, Birth Cohort OR Study, Birth Cohort) |
| #9 | TS=(Case-Control Studies OR Case-Control Study OR Studies, Case-Control OR Study, Case-Control OR Case-Comparison Studies OR Case Comparison Studies OR Case-Comparison Study OR Studies, Case-Comparison OR Study, Case-Comparison OR Case-Compeer Studies OR Studies, Case-Compeer OR Case-Base Studies OR Case Base Studies OR Studies, Case-Base OR Case-Referent Studies OR Case Referent Studies OR Case-Referent Study OR Studies, Case-Referent OR Study, Case-Referent OR Case-Referrent Studies OR Case Referrent Studies OR Case-Referrent Study OR Studies, Case-Referrent OR Study, Case-Referrent OR Case Control Studies OR Case Control Study OR Studies, Case Control OR Study, Case Control OR Matched Case-Control Studies OR Case-Control Studies, Matched OR Case-Control Study, Matched OR Matched Case Control Studies OR Matched Case-Control Study OR Studies, Matched Case-Control OR Study, Matched Case-Control OR Nested Case-Control Studies OR Case-Control Studies, Nested OR Case-Control Study, Nested OR Nested Case Control Studies OR Nested Case-Control Study OR Studies, Nested Case-Control OR Study, Nested Case-Control) |
| #10 | TS=(real-world study) |
| #11 | TS=(Randomized Controlled Trial OR RCT) |
| #12 | #8 OR #9 OR #10 OR #11 |
| #13 | #7 AND #12 |

Table S1 D	Search in Cochrane Library

| #1 | MeSH descriptor: [Proton Pump Inhibitors] explode all trees |
| --- | --- |
| #2 | (Proton Pump Inhibitors OR Pump Inhibitor, Proton OR Inhibitors, Proton Pump OR Inhibitor, Proton Pump OR Proton Pump Inhibitor OR PPIs OR PPI OR ppi):ti,ab,kw (Word variations have been searched) |
| #3 | MeSH descriptor: [Omeprazole] explode all trees |
| #4 | (Omeprazole OR Prilosec OR H 16868 OR H 168 68 OR Sodium, Omeprazole OR Omeprazole Sodium OR Magnesium, Omeprazole OR Omeprazole Magnesium):ti,ab,kw (Word variations have been searched) |
| #5 | MeSH descriptor: [Esomeprazole] explode all trees |
| #6 | (Esomeprazole OR Esomeprazole Sodium OR Strontium, Esomeprazole OR Esomeprazole Strontium OR Esomeprazole Magnesium OR Nexium OR Esomeprazole Strontium Anhydrous OR Esomeprazole Potassium):ti,ab,kw (Word variations have been searched) |
| #7 | MeSH descriptor: [Lansoprazole] explode all trees |
| #8 | (Lansoprazole OR Prezal OR Takepron OR Lansol OR Prevacid OR Opiren OR Ogastro OR Pro Ulco OR Lanzor OR Lansoprazoles OR Lansoprazol OR Zoton OR Ogast OR Agopton OR AG-1749 OR AG 1749 OR AG1749 OR Sodium, Lansoprazole OR Lansoprazole Sodium OR Bamalite OR Ulpax OR Promeco OR Monolitum):ti,ab,kw (Word variations have been searched) |
| #9 | MeSH descriptor: [Pantoprazole] explode all trees |
| #10 | (Pantoprazole OR Protonix OR BY 1023 OR BY-1023 OR BY1023 OR SKF96022 OR SKF 96022 OR SKF-96022 OR SK&F-96022 OR SK&F 96022 OR SK&F96022 OR Pantoprazole Sodium):ti,ab,kw (Word variations have been searched) |
| #11 | MeSH descriptor: [Rabeprazole] explode all trees |
| #12 | (Rabeprazole OR LY307640 OR LY 307640 OR LY-307640 OR E3810 OR E 3810 OR Pariet OR Aciphex OR Rabeprazole Sodium OR Sodium, Rabeprazole OR Dexrabeprazole):ti,ab,kw (Word variations have been searched) |
| #13 | MeSH descriptor: [Dexlansoprazole] explode all trees |
| #14 | (Dexlansoprazole OR Dexilant OR T-168390 OR T 168390 OR T168390 OR TAK-390MR OR TAK390MR OR TAK 390MR ORLansoprazole, R-Isomer OR R-Lansoprazole OR R Lansoprazole OR R-Isomer Lansoprazole OR Lansoprazole, R Isomer OR Dexlansoprazole Sesquihydrate OR TAK-390 OR TAK 390 OR TAK390):ti,ab,kw (Word variations have been searched) |
| #15 | #1 OR #2 OR #3 OR #4 OR #5 OR #6 OR #7 OR #8 OR #9 OR #10 OR #11 OR #12 OR #13 OR #14 |
| #16 | MeSH descriptor: [Fractures, Bone] explode all trees |
| #17 | (Fracture OR Fractures, Bone OR Torsion Fracture OR Spiral Fractures OR Fracture, Torsion OR Spiral Fracture OR Fracture, Spiral OR Fractures, Torsion OR Fractures, Spiral OR Torsion Fractures OR Bone Fracture OR Bone Fractures OR Bones, Broken OR Broken Bone OR Broken Bones OR Bone, Broken OR Fracture, Bone):ti,ab,kw (Word variations have been searched) |
| #18 | MeSH descriptor: [Osteoporosis] explode all trees |
| #19 | (Osteoporosis OR Bone Losses, Age-Related OR Age-Related Bone Losses OR Age-Related Osteoporoses OR Bone Loss, Age-Related OR Age-Related Osteoporosis OR Age Related Osteoporosis OR Age-Related Bone Loss OR Osteoporosis, Age Related OR Osteoporoses, Age-Related OR Bone Loss, Age Related OR Osteoporosis, Age-Related OR Post-Traumatic Osteoporosis OR Osteoporosis, Post Traumatic OR Post-Traumatic Osteoporoses OR Osteoporosis, Post-Traumatic OR Osteoporosis, Involutional OR Senile Osteoporosis OR Osteoporosis, Senile OR Senile Osteoporoses OR Osteoporoses, Senile OR Osteoporoses):ti,ab,kw (Word variations have been searched) |
| #20 | #16 OR #17 OR #18 OR #19 |
| #21 | #15 AND #20 |

**Table S2	Preferred terms (PTs) associated with fracture outcomes and standard medical queries (SMQ) for Osteoporosis/Osteopenia (narrow and broad)**

**Fracture outcomes**

'LUMBAR VERTEBRAL FRACTURE','SPINAL COMPRESSION FRACTURE','SPINAL FRACTURE','HIP FRACTURE','CERVICAL VERTEBRAL FRACTURE','WRIST FRACTURE','RIB FRACTURE','HIP ARTHROPLASTY','FEMUR FRACTURE','PELVIC FRACTURE','FRACTURED SACRUM','FEMORAL NECK FRACTURE','FRACTURE','PATHOLOGICAL FRACTURE','RADIUS FRACTURE','HIP SURGERY','OSTEOPOROTIC FRACTURE','MULTIPLE FRACTURES','STRESS FRACTURE','THORACIC VERTEBRAL FRACTURE','ACETABULUM FRACTURE','ILIUM FRACTURE','FOREARM FRACTURE','OPEN REDUCTION OF FRACTURE','FRACTURE TREATMENT','INTERNAL FIXATION OF FRACTURE','EXTERNAL FIXATION OF FRACTURE','CLOSED FRACTURE MANIPULATION','ATYPICAL FEMUR FRACTURE','OPEN REDUCTION OF SPINAL FRACTURE','SACROILIAC FRACTURE','SACROILIAC FUSION','PUBIC RAMI FRACTURE','FRACTURED ISCHIUM','OSTEOSYNTHESIS','REVISION OF INTERNAL FIXATION','PUBIS FRACTURE','SUBCHONDRAL INSUFFICIENCY FRACTURE',

**Osteoporosis/Osteopenia (narrow)**

'OSTEOPOROSIS','BONE DENSITY DECREASED','OSTEOPENIA','OSTEOPOROTIC FRACTURE','BONE FORMATION DECREASED','OSTEOPOROSIS POSTMENOPAUSAL','RESORPTION BONE INCREASED','BONE MARROW OEDEMA SYNDROME','SENILE OSTEOPOROSIS','BONE LOSS',

**Osteoporosis/Osteopenia (broad)**

'LUMBAR VERTEBRAL FRACTURE','SPINAL COMPRESSION FRACTURE','OSTEOPOROSIS','BONE DENSITY DECREASED','SPINAL FRACTURE','HIP FRACTURE','CERVICAL VERTEBRAL FRACTURE','WRIST FRACTURE','RIB FRACTURE','HIP ARTHROPLASTY','FEMUR FRACTURE','PELVIC FRACTURE','FRACTURED SACRUM','FEMORAL NECK FRACTURE','BODY HEIGHT DECREASED','FRACTURE','PATHOLOGICAL FRACTURE','RADIUS FRACTURE','OSTEOPENIA','HIP SURGERY','OSTEOPOROTIC FRACTURE','MULTIPLE FRACTURES','KYPHOSCOLIOSIS','STRESS FRACTURE','SPINAL DEFORMITY','KYPHOSIS','THORACIC VERTEBRAL FRACTURE','ACETABULUM FRACTURE','ILIUM FRACTURE','BONE FORMATION DECREASED','FOREARM FRACTURE','BODY HEIGHT BELOW NORMAL','POST-TRAUMATIC OSTEOPOROSIS','OSTEOCALCIN INCREASED','OPEN REDUCTION OF FRACTURE','WRIST SURGERY','BONE METABOLISM DISORDER','OSTEOPOROSIS POSTMENOPAUSAL','FRACTURE TREATMENT','INTERNAL FIXATION OF FRACTURE','BODY HEIGHT ABNORMAL','VERTEBROPLASTY','RESORPTION BONE INCREASED','DEOXYPYRIDINOLINE URINE INCREASED','EXTERNAL FIXATION OF FRACTURE','BONE MARROW OEDEMA SYNDROME','SENILE OSTEOPOROSIS','C-TELOPEPTIDE','CLOSED FRACTURE MANIPULATION','C-TELOPEPTIDE INCREASED','BONE RESORPTION TEST ABNORMAL','PYRIDINOLINE URINE INCREASED','BONE DENSITY ABNORMAL','BONE FORMATION TEST ABNORMAL','BONE LOSS','ATYPICAL FEMUR FRACTURE','BONE RESORPTION TEST','OPEN REDUCTION OF SPINAL FRACTURE','OSTEOPOROSIS PROPHYLAXIS','SACROILIAC FRACTURE','VERTEBRAL BODY REPLACEMENT','BONE METABOLISM BIOCHEMICAL MARKER INCREASED','SERUM PROCOLLAGEN TYPE I N-TERMINAL PROPEPTIDE DECREASED','SHORT STATURE','TARTRATE-RESISTANT ACID PHOSPHATASE DECREASED','SACROILIAC FUSION','BONE DEMINERALISATION','CARPECTOMY','N-TELOPEPTIDE INCREASED','N-TELOPEPTIDE ABNORMAL','PUBIC RAMI FRACTURE','FRACTURED ISCHIUM','OSTEOSYNTHESIS','N-TELOPEPTIDE URINE INCREASED','REVISION OF INTERNAL FIXATION','PUBIS FRACTURE','BONE DECALCIFICATION','N-TELOPEPTIDE URINE ABNORMAL','SUBCHONDRAL INSUFFICIENCY FRACTURE',

**Table S3	The calculation procedure for the ROR, PRR and IC**

| Calculation Procedure | Threshold for significance |
| --- | --- |
|      | Significance was defined as ROR025 > 1 with at least 4 cases |
|   SE(lnPRR)=  =   | Significance was defined as PRR025 > 1with at least 4 cases |
| *α_i_*=*β_j_*=1,*α*=*β*=2,*γ_ij_*=1  *N*＝*a*+*b*+*c*+*d* $\text{IC}=\log_{2}\frac{a\text{(}a+b+c+d\text{)}}{\text{(}a+b\text{)(}a+c\text{)}}$ $\gamma\text{=}\gamma_{_{ij}}\frac{\text{(}N\text{+}\alpha\text{)(}N\text{+}\beta\text{)}}{\text{(}a+b\text{+}\alpha i\text{)(}a+c\text{+}\beta j\text{)}}$ $\text{E(IC)}=\log_{2}^{^{\frac{\text{(}a\text{+}\gamma_{ij}\text{)(}N\text{+}\alpha\text{)(}N\text{+}\beta\text{)}}{\text{(}N\text{+}\gamma\text{)(}a\text{+}b\text{+}\alpha_{i}\text{)(}a\text{+}c\text{+}\beta_{j}\text{)}}}}$       V(IC)= $\text{SD}=\sqrt{\text{V(IC)}}$   IC025=E(IC)-2SD | Significance was defined as IC025 > 0, with at least 4 cases |

**ROR, Reporting Odds Ratio; PRR, Proportional Reporting Ratio; IC, Information Component.**

**Table S4	Five hierarchical models of disproportionality analysis (DA)**

| Model | Description |
| --- | --- |
| #1 | No restrictions applied. |
| #2 | Set 'primary suspect' and report Adverse Drug Reactions (ADRs) by healthcare professionals (including MDs, Pharmacists, and Other Health Professionals). |
| #3 | Exclude patients with ADR risk factors (diseases) from Model 2.  Including various endocrine system diseases such as hypogonadism, diabetes, hyperthyroidism, as well as rheumatologic and autoimmune diseases, gastrointestinal disorders, hematological diseases, neuromuscular diseases, and chronic liver, kidney, and cardiopulmonary diseases. |
| #4 | Exclude patients with ADR risk factors (drugs) from Model 3.  Including 'GLUCOCORTICOID','PREDNISONE','PREDNISOLONE','METHYLPREDNISOLONE','DEXAMETHASONE','PHENYTOIN','CARBAMAZEPINE','PHENOBARBITAL','VALPROATE','TOPIRAMATE','ANASTROZOLE','LETROZOLE','EXEMESTANE','LEUPRORELIN','GOSERELIN','TRIPTORELIN','DEGARELIX','TENOFOVIR DISOPROXIL FUMARATE','PIOGLITAZONE','ROSIGLITAZONE' |
| #5 | Exclude all cases with concurrent use of other acid-suppressing drugs from Model 4. |

**Table S5	Baseline characteristics of included studies**

| **Author, year** | **Study design** | **Study period** | **Country** | **Population** | **No. of participants** | **Age, years** | **Male, %** | **Follow-up duration** | **Fracture outcome** | **PPI treatment/comparator** | **Major adjusted confounders** |
| --- | --- | --- | --- | --- | --- | --- | --- | --- | --- | --- | --- |
| Gingold-Belfer et al., 2023 | Co (retrospective) | 2002-2016 | Israel | Community-dwelling adults aged ≥65 years registered with Clalit Health Services; persons with an osteoporotic fracture before 2003 or who died in 2002 were excluded. | 46,805 | 83.4 ± 6.4 | 41.9 | Median 12 years (mean 10.4 ± 4.8); followed to fracture, death, or 31 Dec 2016. | Osteoporotic hip, vertebral, or wrist fracture recorded in hospital, outpatient, or primary-care records; diagnoses were based on clinical and imaging evaluations. | Omeprazole  No PPI use | Age; sex; Crohn disease; depression; osteoporosis; rheumatoid arthritis; arthropathy; disability; chronic liver disease; multiple myeloma; chronic obstructive pulmonary disease. |
| Labenz et al., 2020 | C-C (nested) | 2010-2019 | Germany | Adults aged ≥18 years with liver cirrhosis in 1,178 general practices; patients with a first recorded bone fracture were compared with cirrhotic controls without fracture. | 12,030 | 63.8 ± 12.9 | 59.6 | Mean observation: 7.9 ± 5.7 years in cases and 7.8 ± 5.8 years in controls; ≥12-month pre-index observation required; cumulative PPI exposure assessed during the preceding 5 years. | First recorded bone fracture of shoulder/arm, ribs/sternum/thoracic spine, lower leg/foot/toe, femur, lumbar spine/pelvis, or other/unspecified sites, identified by ICD-10 codes. | Pantoprazole  Omeprazole  No PPI use | Age; sex; cirrhosis etiology and compensated/decompensated status; diabetes; cardiovascular, pulmonary, cerebrovascular, and chronic kidney diseases; osteoporosis; cancer; dementia; alcohol-related disorders; beta-blockers; opioids; antidepressants; antipsychotics; hypnotics/anxiolytics; corticosteroids; bisphosphonates. |
| van der Hoorn et al., 2015 | Co (prospective) | 2003-2011 | Australia | Older women (born 1921-1926) in the Australian Longitudinal Study on Women's Health; hospital-linked fracture data were available for four states/territories. | 4,432 overall; 2,734 in fracture analysis | 78.2 ± 1.5 | 0 | Average 7.6 years for fractures; from 1 Jan 2003 to first fracture, death, or 30 Jun 2011. | Any fracture recorded with ICD-10 codes in linked admitted-patient hospital datasets. | Rabeprazole  Pantoprazole  Omeprazole  Lansoprazole  Esomeprazole  No PPI use | Age; area of residence; BMI; number of chronic conditions; physical functioning; thyroid hormones; high-dose glucocorticoids; aromatase inhibitors; selective serotonin reuptake inhibitors. |
| Torvinen-Kiiskinen et al., 2018 | C-C (nested) | 2005-2011 | Finland | Community-dwelling persons with clinically verified Alzheimer disease; incident hip-fracture cases were matched to controls, and persons with hip fracture before Alzheimer diagnosis were excluded. | 24,053 | Cases 84.1 ± 6.0; controls 84.0 ± 5.8 | 25.0 | Case-control exposure window: current use 0-30 days and past use 31-90 days before index; cumulative PPI use assessed over the preceding 10 years. | Incident hip fracture identified in the Hospital Discharge Register: femoral neck (S72.0), pertrochanteric (S72.1), or subtrochanteric fracture (S72.2). | Rabeprazole  Pantoprazole  Omeprazole  Lansoprazole  Esomeprazole  No PPI use | Matched on age, sex, and time since Alzheimer diagnosis; adjusted for socioeconomic position, hospital days, cardiovascular disease, diabetes, asthma/COPD, glaucoma, rheumatoid arthritis, epilepsy, previous fracture, stroke, cancer, psychiatric disorders, substance abuse, oral corticosteroids, bisphosphonates, antipsychotics, antidepressants, benzodiazepines, opioids, antithrombotics, H2 blockers, and Alzheimer medications. |
| Adams et al., 2014 | C-C | 1991-2006 (exposure); 1997-2006 (fractures) | United States | Men aged ≥45 years enrolled in Kaiser Permanente Southern California; incident hip-fracture cases with no prior hip fracture were matched to men without hip fracture. | 13,548 | ≥45; mean/median not reported | 100 | PPI exposure assessed from 1991 to the index date (up to 15 years); comorbidities assessed during the 2-year pre-index look-back period. | Incident hip fracture identified from electronic records/claims using ICD-9-CM 820-820.9; cases with evidence of a previous hip fracture were excluded. | Pantoprazole  Omeprazole  No PPI use | Matched on age, race/ethnicity, medical center, and health-plan membership at index; adjusted for continuous Charlson comorbidity score. |
| Cea Soriano et al., 2014 | C-C (nested in cohort) | 2000-2008 | United Kingdom | Patients aged 40-89 years in The Health Improvement Network primary-care database; validated hip-fracture cases were sampled with incidence-density controls. | 30,958 (10,958 cases; 20,000 controls) | 40-89; mean not reported | 25.0 | Mean 5.5 years in the source cohort; exposure categories used prescription end dates 0-30, 31-90, 91-365, or >365 days before index. | First recorded Read-code diagnosis of hip fracture; cases were validated by record review/physician questionnaires, and fractures related to road traffic accidents were excluded. | Rabeprazole  Pantoprazole  Omeprazole  Lansoprazole  Esomeprazole  No PPI use | Age; sex; index year; primary-care visits; specialist referrals; hospital admissions; BMI; cardiovascular disease; dementia; GERD; osteoporosis; peptic ulcer; cerebrovascular disease; rheumatoid arthritis; anemia; Charlson score; antidepressants; NSAIDs; oral steroids; statins; vitamin D plus calcium. |
| Lee et al., 2013 | C-C | Jan 2005-Jun 2006 | South Korea | National claims data for adults aged ≥65 years; incident hip-fracture cases were matched with controls without fracture by age, sex, and osteoporosis. | 123,352 | 77.7 ± 7.3 | 26.6 | Exposure assessed from 1 Jan 2005 to index (maximum 18 months); current use 0-30 days, recent use 31-90 days, and past use ≥91 days before index. | Incident femoral neck (S72.0), pertrochanteric (S72.1), or subtrochanteric hip fracture (S72.2); hip-fracture diagnoses in the first 6 months of 2005 were excluded. | Rabeprazole  Pantoprazole  Omeprazole  Lansoprazole  Esomeprazole  No PPI use | Matched on age, sex, and osteoporosis; adjusted for diabetes, thyroid disease, hyperparathyroidism, Cushing syndrome, asthma, COPD, chronic renal failure, heart failure, dementia, stroke, corticosteroids, warfarin, antiepileptics, antidepressants, benzodiazepines, anti-Parkinson drugs, H2 blockers, bisphosphonates, and raloxifene. |
| Reyes et al., 2013 | C-C | 2007-2010 | Catalonia, Spain | Patients aged ≥50 years attending six primary-care centers; cases had a low-trauma fragility hip fracture and controls had no recorded hip fracture. | 1,056 | 82.0 ± 8.8 | 23.0 | Case-control exposure window: clinical, therapeutic, and PPI prescription history during the 5 years before index hip fracture. | Fragility hip fracture caused by a fall no higher than the person's height; neoplastic fractures excluded; identified using ICD-9 hip/femur fracture or hip-prosthesis codes. | Rabeprazole  Pantoprazole  Omeprazole  Lansoprazole  Esomeprazole  No PPI use | Matched on age and sex; adjusted for alcohol intake, schizophrenia, dementia, osteoporosis, pernicious anemia, depression, calcium, vitamin D, corticosteroids, antiepileptic drugs, insulin, and SSRI/non-SSRI antidepressants. |
| Roux et al., 2008 | Co (prospective) | 1999-2007 | Multiple European countries | Ambulatory postmenopausal women aged 55-79 years recruited from random population samples in five European centers (OPUS substudy). | 1,211 | 65.8 ± 6.6 | 0 | Mean 6.1 years; vertebral radiographs obtained at baseline and final follow-up. | Incident vertebral fracture assessed centrally on standardized radiographs using semiquantitative reading, excluding nonosteoporotic deformities; nonvertebral fractures were collected by questionnaire. | Omeprazole  No PPI use | Stepwise model considered baseline variables differing at P<0.20; the final model retained age >65 years, prevalent vertebral fracture, and lumbar-spine T-score ≤-2.5. |

**For case-control studies, follow-up duration refers to the exposure assessment window or look-back period when applicable. C-C, case-control study; Co, cohort study; PPI, proton pump inhibitor.**

**Table S6	Risk of bias in included observational studies (by Newcastle-Ottawa Scale)**

| **Cohort studies** | | | | | | | | | |
| --- | --- | --- | --- | --- | --- | --- | --- | --- | --- |
|  | **Selection** | | | | **Comparability** | **Outcome** | | |  |
| **Author, year** | **Representativeness of the exposed cohort** | **Selection of the non-exposed cohort** | **Ascertainment of exposure** | **Concealed demonstration of outcomes before exposure** | **Comparability of cohorts on the basis of design** | **Assessment of outcome** | **Follow-up long enough for outcomes to occur** | **Adequacy of follow up for cohorts** | **Total score** |
| G-Belfer (2023) | ★ | ★ | ★ | ★ | ★★ | ★ | ★ | ☆ | 8 |
| Hoorn (2015) | ★ | ★ | ★ | ★ | ★ | ★ | ★ | ☆ | 7.5 |
| Roux (2009) | ★ | ★ | ☆ | ★ | ★★ | ★ | ★ | ☆ | 7 |
| **Case-control studies** | | | | | | | | | |
|  | **Selection** | | | | **Comparability** | **Outcome** | | |  |
| **Author, year** | **Adequate case definition** | **Representativeness of the cases** | **Selection of controls** | **Definition of controls** | **Comparability of cases and controls on the basis of design** | **Ascertainment of exposure** | **Same ascertainment for cases and controls** | **Non-response rate** | **Total score** |
| Labenz (2020) | ★ | ★ | ★ | ★ | ★★ | ☆ | ★ | ☆ | 7 |
| T-Kiiskinen (2018) | ★ | ★ | ★ | ★ | ★★ | ★ | ★ | ☆ | 8 |
| Adams (2014) | ★ | ★ | ★ | ★ | ★☆ | ★ | ★ | ☆ | 7 |
| Soriano (2014) | ★ | ★ | ★ | ★ | ★★ | ★ | ★ | ☆ | 8 |
| Lee (2013) | ★ | ★ | ★ | ★ | ☆☆ | ★ | ★ | ☆ | 6 |
| Reyes (2013) | ★ | ★ | ★ | ★ | ★★ | ★ | ★ | ☆ | 8 |

**NOS has a total maximum score of 9: Maximum scores of 4 in Selection, 2 in Comparability, and 3 in Outcome. Studies scoring 7–9 were considered to have good quality (low risk of bias), 4–6 fair quality (moderate risk), and 0–3 poor quality (high risk). The scoring symbols are defined as follows: full star (★) = 1 point, half-star ( ) = 0.5 point, and empty star (☆) = 0 points in each category**

**Table S7	SUCRA probability evaluations and P-score of NMA**

| Fracture of any site | | | |
| --- | --- | --- | --- |
| Treatment | SUCRA (%) | Mean Rank | P-score |
| Omeprazole | 86.3 | 1.7 | 0.864 |
| Rabeprazole | 76.1 | 2.2 | 0.762 |
| Pantoprazole | 69.5 | 2.5 | 0.693 |
| Lansoprazole | 45.1 | 3.7 | 0.453 |
| Esomeprazole | 21.8 | 4.9 | 0.217 |
| No PPI use | 1.0 | 5.9 | 0.011 |
| Hip fracture | | | |
| Treatment | SUCRA (%) | Mean Rank | P-score |
| Omeprazole | 79.2 | 2.0 | 0.791 |
| Rabeprazole | 78.2 | 2.1 | 0.780 |
| Pantoprazole | 71.3 | 2.4 | 0.714 |
| Lansoprazole | 47.3 | 3.6 | 0.475 |
| Esomeprazole | 21.4 | 4.9 | 0.214 |
| No PPI use | 2.6 | 5.9 | 0.026 |

**SUCRA=surface under the cumulative ranking curve; NMA, network meta-analysis**

**Table S8	Sensitivity Analysis of NMA**

| Fracture of any site | | | | Hip fracture | | | |
| --- | --- | --- | --- | --- | --- | --- | --- |
| Treatment | SUCRA (%) | Mean Rank | P-score | Treatment | SUCRA(%) | Mean Rank | P-score |
| Omitting G-Belfer (2023) | | | | Omitting T-Kiiskinen (2018) | | | |
| Omeprazole | 86.4 | 1.7 | 0.865 | Pantoprazole | 77.5 | 2.1 | 0.774 |
| Rabeprazole | 75.5 | 2.2 | 0.756 | Omeprazole | 74.7 | 2.3 | 0.745 |
| Pantoprazole | 69.4 | 2.5 | 0.692 | Rabeprazole | 69.3 | 2.5 | 0.695 |
| Lansoprazole | 45.7 | 3.7 | 0.457 | Lansoprazole | 52.5 | 3.4 | 0.526 |
| Esomeprazole | 22.0 | 4.9 | 0.219 | Esomeprazole | 21.1 | 4.9 | 0.211 |
| No PPI use | 1.1 | 5.9 | 0.011 | No PPI use | 4.9 | 5.8 | 0.049 |
| Omitting Labenz (2020) | | | | Omitting Adams (2014) | | | |
| Omeprazole | 82.0 | 1.9 | 0.821 | Omeprazole | 78.2 | 2.1 | 0.782 |
| Rabeprazole | 80.3 | 2.0 | 0.803 | Rabeprazole | 74.9 | 2.3 | 0.749 |
| Pantoprazole | 69.7 | 2.5 | 0.696 | Pantoprazole | 74.0 | 2.3 | 0.741 |
| Lansoprazole | 45.7 | 3.7 | 0.458 | Lansoprazole | 47.8 | 3.6 | 0.478 |
| Esomeprazole | 21.1 | 4.9 | 0.210 | Esomeprazole | 22.4 | 4.9 | 0.224 |
| No PPI use | 1.3 | 5.9 | 0.013 | No PPI use | 2.6 | 5.9 | 0.026 |
| Omitting Hoorn (2015) | | | | Omitting Lee (2013) | | | |
| Omeprazole | 86.2 | 1.7 | 0.863 | Omeprazole | 95.2 | 1.2 | 0.953 |
| Rabeprazole | 73.9 | 2.3 | 0.740 | Lansoprazole | 72.2 | 2.4 | 0.722 |
| Pantoprazole | 69.8 | 2.5 | 0.696 | Pantoprazole | 55.5 | 3.2 | 0.557 |
| Lansoprazole | 45.9 | 3.7 | 0.460 | Rabeprazole | 41.4 | 3.9 | 0.414 |
| Esomeprazole | 22.5 | 4.9 | 0.225 | Esomeprazole | 31.8 | 4.4 | 0.317 |
| No PPI use | 1.6 | 5.9 | 0.017 | No PPI use | 3.8 | 5.8 | 0.038 |
| Omitting T-Kiiskinen (2018) | | | | Omitting Reyes (2013) | | | |
| Omeprazole | 83.7 | 1.8 | 0.837 | Rabeprazole | 80.8 | 2.0 | 0.809 |
| Pantoprazole | 74.0 | 2.3 | 0.737 | Pantoprazole | 75.4 | 2.2 | 0.753 |
| Rabeprazole | 69.4 | 2.5 | 0.695 | Omeprazole | 74.7 | 2.3 | 0.746 |
| Lansoprazole | 49.5 | 3.5 | 0.497 | Lansoprazole | 45.7 | 3.7 | 0.458 |
| Esomeprazole | 21.3 | 4.9 | 0.211 | Esomeprazole | 19.7 | 5.0 | 0.197 |
| No PPI use | 2.1 | 5.9 | 0.022 | No PPI use | 3.6 | 5.8 | 0.036 |
| Omitting Adams (2014) | | | | Omitting Soriano (2014) | | | |
| Omeprazole | 86.2 | 1.7 | 0.863 | Rabeprazole | 91.3 | 1.4 | 0.915 |
| Rabeprazole | 73.9 | 2.3 | 0.742 | Pantoprazole | 72.9 | 2.4 | 0.729 |
| Pantoprazole | 71.9 | 2.4 | 0.717 | Omeprazole | 69.5 | 2.5 | 0.693 |
| Lansoprazole | 44.9 | 3.8 | 0.450 | Lansoprazole | 39.4 | 4.0 | 0.395 |
| Esomeprazole | 22.1 | 4.9 | 0.221 | Esomeprazole | 21.2 | 4.9 | 0.211 |
| No PPI use | 0.9 | 6.0 | 0.009 | No PPI use | 5.6 | 5.7 | 0.057 |
| Omitting Lee (2013) | | | |  |  |  |  |
| Omeprazole | 95.3 | 1.2 | 0.954 |  |  |  |  |
| Lansoprazole | 61.8 | 2.9 | 0.616 |  |  |  |  |
| Pantoprazole | 60.6 | 3.0 | 0.605 |  |  |  |  |
| Rabeprazole | 52.8 | 3.4 | 0.531 |  |  |  |  |
| Esomeprazole | 28.6 | 4.6 | 0.286 |  |  |  |  |
| No PPI use | 0.9 | 6.0 | 0.009 |  |  |  |  |
| Omitting Reyes (2013) | | | |  |  |  |  |
| Omeprazole | 83.9 | 1.8 | 0.840 |  |  |  |  |
| Rabeprazole | 78.3 | 2.1 | 0.782 |  |  |  |  |
| Pantoprazole | 71.8 | 2.4 | 0.717 |  |  |  |  |
| Lansoprazole | 43.5 | 3.8 | 0.435 |  |  |  |  |
| Esomeprazole | 21.1 | 4.9 | 0.210 |  |  |  |  |
| No PPI use | 1.4 | 5.9 | 0.015 |  |  |  |  |
| Omitting Soriano (2014) | | | |  |  |  |  |
| Rabeprazole | 88.8 | 1.6 | 0.888 |  |  |  |  |
| Omeprazole | 78.1 | 2.1 | 0.782 |  |  |  |  |
| Pantoprazole | 69.4 | 2.5 | 0.693 |  |  |  |  |
| Lansoprazole | 38.3 | 4.1 | 0.384 |  |  |  |  |
| Esomeprazole | 23.0 | 4.9 | 0.229 |  |  |  |  |
| No PPI use | 2.4 | 5.9 | 0.024 |  |  |  |  |
| Omitting Roux (2009) | | | |  |  |  |  |
| Omeprazole | 85.0 | 1.7 | 0.852 |  |  |  |  |
| Rabeprazole | 76.9 | 2.2 | 0.768 |  |  |  |  |
| Pantoprazole | 69.9 | 2.5 | 0.699 |  |  |  |  |
| Lansoprazole | 45.4 | 3.7 | 0.454 |  |  |  |  |
| Esomeprazole | 21.6 | 4.9 | 0.216 |  |  |  |  |
| No PPI use | 1.1 | 5.9 | 0.012 |  |  |  |  |
| Omitting Adams (2014) and Labenz (2020) | | | |  |  |  |  |
| Omeprazole | 81.2 | 1.9 | 0.812 |  |  |  |  |
| Rabeprazole | 77.3 | 2.1 | 0.773 |  |  |  |  |
| Pantoprazole | 73.5 | 2.3 | 0.735 |  |  |  |  |
| Lansoprazole | 45.5 | 3.7 | 0.454 |  |  |  |  |
| Esomeprazole | 21.4 | 4.9 | 0.215 |  |  |  |  |
| No PPI use | 1.2 | 5.9 | 0.012 |  |  |  |  |

**Yellow highlighting denotes a change in the treatment's ranking position upon exclusion of the respective study, reflecting the influence of individual studies. Overall rankings remained largely stable. SUCRA=surface under the cumulative ranking curve; NMA, network meta-analysis**

**Table S9	Results of disproportionality analyses for PPIs and Bone-Related Outcomes (ROR, PRR and IC)**

| **Drug** | **ROR ( 95% CI)** | **PRR ( 95% CI)** | **IC025** |
| --- | --- | --- | --- |
| Osteoporosis/osteopenia (SMQ)(broad definition) | | | |
| Omeprazole | 1.33（1.12-1.58） | 1.33（1.12-1.57） | 0.15 |
| Lansoprazole | 1.15（0.87-1.51） | 1.15（0.87-1.5） | -0.21 |
| Pantoprazole | 1.02（0.84-1.25） | 1.02（0.84-1.25） | -0.26 |
| Rabeprazole | 0.89（0.49-1.61） | 0.89（0.49-1.61） | -0.99 |
| Esomeprazole | 1.02（0.79-1.32） | 1.02（0.79-1.32） | -0.35 |
| Osteoporosis/osteopenia (SMQ)(narrow definition) | | | |
| Omeprazole | 2.22（1.45-3.42） | 2.22（1.45-3.41） | 0.45 |
| Lansoprazole | 1.65（0.78-3.46） | 1.65（0.79-3.46） | -0.42 |
| Pantoprazole | 1.01（0.53-1.95） | 1.01（0.53-1.95） | -0.9 |
| Rabeprazole | 2.61（0.84-8.09） | 2.6（0.84-8.07） | -0.55 |
| Esomeprazole | 2.44（1.41-4.2） | 2.43（1.41-4.2） | 0.36 |
| Fracture of any site | | | |
| Omeprazole | 1.25（1.03-1.51） | 1.25（1.03-1.51） | 0.04 |
| Lansoprazole | 1.15（0.85-1.54） | 1.15（0.86-1.53） | -0.24 |
| Pantoprazole | 1.08（0.88-1.34） | 1.08（0.88-1.33） | -0.19 |
| Rabeprazole | 0.75（0.37-1.5） | 0.75（0.37-1.5） | -1.34 |
| Esomeprazole | 0.89（0.66-1.2） | 0.89（0.66-1.2） | -0.59 |

**ROR, Reporting Odds Ratio; PRR, Proportional Reporting Ratio; IC, Information Component; 95% CI, 95% Confidence Interval; SMQ, Standardised MedDRA Query**

**Table S10	Subgroup Analysis of disproportionality analysis (DA)**

| **Drug** | **No. of cases; ROR (95% CI)** | | | | |
| --- | --- | --- | --- | --- | --- |
|  | **Age（years）** | | | **Sex** | |
|  | **65-74** | **75-84** | **≥85** | **Male** | **Female** |
| **Osteoporosis/osteopenia (SMQ) (broad definition)** | | | | | |
| Omeprazole | 34; 0.98（0.7-1.37） | 58; 1.28（0.99-1.66） | **41; 1.63（1.19-2.22）** | 47; 1.24（0.93-1.65） | **168; 1.54（1.32-1.79）** |
| Lansoprazole | 10; 0.67（0.36-1.24） | 16; 0.85（0.52-1.39） | **26; 1.65（1.12-2.44）** | 21; 1.47（0.96-2.26） | 60; 1.27（0.98-1.63） |
| Pantoprazole | 27; 0.77（0.53-1.12） | 49; 1.24（0.94-1.65） | 21; 0.84（0.55-1.3） | **52; 1.49（1.13-1.95）** | 87; 0.94（0.76-1.16） |
| Rabeprazole | 5; 1.13（0.47-2.72） | 5; 0.88（0.36-2.12） | 1; 0.36（0.05-2.55） | 6; 1.46（0.66-3.26） | 13; 0.94（0.55-1.63） |
| Esomeprazole | 22; 1.07（0.71-1.63） | 22; 0.91（0.6-1.39） | 14; 0.98（0.58-1.66） | **56; 2.76（2.12-3.59）** | **81; 1.26（1.01-1.56）** |
| **Osteoporosis/osteopenia (SMQ) (narrow definition)** | | | | | |
| Omeprazole | 7; 1.58（0.75-3.33） | **11; 3（1.66-5.45）** | 3; 2.55（0.81-7.96） | 9; 1.9（0.99-3.67） | **35; 2.06（1.47-2.87）** |
| Lansoprazole | 1; 0.53（0.07-3.73） | 1; 0.67（0.09-4.8） | **5; 6.97（2.87-16.95）** | 3; 1.68（0.54-5.23） | 12; 1.64（0.93-2.89） |
| Pantoprazole | 6; 1.35（0.6-3.01） | 3; 0.96（0.31-2.98） | （-） | 4; 0.92（0.35-2.46） | 18; 1.26（0.79-2） |
| Rabeprazole | 3; 5.35（1.72-16.64） | （-） | （-） | 2; 3.92（0.98-15.7） | 4; 1.88（0.71-5.02） |
| Esomeprazole | **7; 2.69（1.28-5.65）** | 4; 2.09（0.78-5.6） | 2; 3.05（0.76-12.28） | **20; 7.96（5.12-12.37）** | **30; 3（2.09-4.29）** |
| **Fracture of any site** | | | | | |
| Omeprazole | 24; 0.83（0.56-1.24） | 47; 1.18（0.88-1.57） | **37; 1.6（1.15-2.21）** | 35; 1.12（0.8-1.56） | **129; 1.48（1.24-1.76）** |
| Lansoprazole | 9; 0.73（0.38-1.4） | 15; 0.9（0.54-1.5） | 21; 1.45（0.94-2.23） | 18; 1.53（0.96-2.43） | 43; 1.13（0.84-1.53） |
| Pantoprazole | 21; 0.72（0.47-1.11） | **47; 1.35（1.01-1.8）** | 21; 0.92（0.6-1.41） | **48; 1.66（1.25-2.21）** | 68; 0.92（0.72-1.16） |
| Rabeprazole | 2; 0.55（0.14-2.18） | 5; 1（0.41-2.4） | 1; 0.39（0.05-2.77） | 3; 0.89（0.29-2.75） | 9; 0.82（0.42-1.57） |
| Esomeprazole | 15; 0.88（0.53-1.47） | 17; 0.8（0.5-1.29） | 12; 0.91（0.52-1.61） | **35; 2.09（1.5-2.91）** | 49; 0.95（0.72-1.25） |

**The bolded ones are n > 4 and ROR025 > 1. ROR, Reporting Odds Ratio; SMQ, Standardised MedDRA Query; 95% CI, 95% Confidence Interval**

# 2 Supplementary Figures

# **Supplementary Figure S1.** Inconsistency tests between direct and indirect evidence (Fracture of any site).

**Supplementary Figure S2.** Inconsistency tests between direct and indirect evidence (Hip fracture).

**Supplementary Figure S3.** Funnel plot and Egger's test for the analysis endpoints.

**Supplementary Figure S4.** Forest plot of conventional meta-analysis (Fracture of any site).

**Supplementary Figure S5.** Forest plot of conventional meta-analysis (Hip Fracture).

**Supplementary Figure S6.**League Charts of network meta-analysis after exclusion of the Lee et al. 2013 study. Each cell shows the effect size, presented as OR with 95% CIs. Results of fracture of any site are reported in the lower left part of the table (column versus row) and results of hip fracture are reported in the upper right part of the table(row versus column). An OR greater than 1 indicates a higher risk in the comparison, whereas an OR less than 1 indicates a lower risk. For example, the cell at the column "Omeprazole" and row "No PPI use" shows an OR of 1.53 (95%CI 1.38-1.68), indicating a 53% increased risk of any-site fracture in omeprazole users compared with non-PPI users. Bold ORs indicate statistically significant results. OR=Odds Ratio.

**Supplementary Figure S7.** Results of disproportionality analyses for PPIs in the FAERS database (Model 1).

**Supplementary Figure S8.** Results of disproportionality analyses for PPIs in the FAERS database (Model 2).

**Supplementary Figure S9.** Results of disproportionality analyses for PPIs in the FAERS database (Model 3).

**Supplementary Figure S10.** Results of disproportionality analyses for PPIs in the FAERS database (Model 4).

**References**

ADAMS, A. L., BLACK, M. H., ZHANG, J. L., SHI, J. M. & JACOBSEN, S. J. (2014). Proton-pump inhibitor use and hip fractures in men: a population-based case-control study. *Annals of Epidemiology.* 24(4)**,** 286-290. 10.1016/j.annepidem.2014.01.004

CEA SORIANO, L., RUIGóMEZ, A., JOHANSSON, S. & GARCíA RODRíGUEZ, L. A. (2014). Study of the Association Between Hip Fracture and Acid‐Suppressive Drug Use in a UK Primary Care Setting. *Pharmacotherapy: The Journal of Human Pharmacology and Drug Therapy.* 34(6)**,** 570-581. 10.1002/phar.1410

GINGOLD‐BELFER, R., BELOOSESKY, Y., AMARA, A., SHARON, E., BOLTIN, D., KOREN‐MORAG, N., et al. (2023). Different effects of chronic omeprazole use on osteoporotic fractures rate in the elderly. *British Journal of Clinical Pharmacology.* 89(12)**,** 3539-3550. 10.1111/bcp.15847

LABENZ, C., WöRNS, M. A., ADARKWAH, C. C., GALLE, P. R., SCHATTENBERG, J. M. & KOSTEV, K. (2020). Proton pump inhibitors increase risk of bone fractures in men with cirrhosis: a population‐based study. *Alimentary Pharmacology & Therapeutics.* 52(6)**,** 1042-1050. 10.1111/apt.16008

LEE, J., YOUN, K., CHOI, N.-K., LEE, J.-H., KANG, D., SONG, H.-J., et al. (2013). A population-based case–control study: proton pump inhibition and risk of hip fracture by use of bisphosphonate. *Journal of Gastroenterology.* 48(9)**,** 1016-1022. 10.1007/s00535-012-0722-9

REYES, C., FORMIGA, F., CODERCH, M., HOYO, J., FERRIZ, G., CASANOVAS, J., et al. (2013). Use of proton pump inhibitors and risk of fragility hip fracture in a Mediterranean region. *Bone.* 52(2)**,** 557-561. 10.1016/j.bone.2012.09.028

ROUX, C., BRIOT, K., GOSSEC, L., KOLTA, S., BLENK, T., FELSENBERG, D., et al. (2008). Increase in Vertebral Fracture Risk in Postmenopausal Women Using Omeprazole. *Calcified Tissue International.* 84(1)**,** 13-19. 10.1007/s00223-008-9188-4

TORVINEN‐KIISKINEN, S., TOLPPANEN, A. M., KOPONEN, M., TANSKANEN, A., TIIHONEN, J., HARTIKAINEN, S., et al. (2018). Proton pump inhibitor use and risk of hip fractures among community‐dwelling persons with Alzheimer's disease—a nested case‐control study. *Alimentary Pharmacology & Therapeutics.* 47(8)**,** 1135-1142. 10.1111/apt.14589

VAN DER HOORN, M. M. C., TETT, S. E., DE VRIES, O. J., DOBSON, A. J. & PEETERS, G. M. E. E. (2015). The effect of dose and type of proton pump inhibitor use on risk of fractures and osteoporosis treatment in older Australian women: A prospective cohort study. *Bone.* 81675-682. 10.1016/j.bone.2015.08.024

ADAMS, A. L., BLACK, M. H., ZHANG, J. L., SHI, J. M. & JACOBSEN, S. J. (2014). Proton-pump inhibitor use and hip fractures in men: a population-based case-control study. Annals of Epidemiology. 24(4), 286-290. 10.1016/j.annepidem.2014.01.004

CEA SORIANO, L., RUIGóMEZ, A., JOHANSSON, S. & GARCíA RODRíGUEZ, L. A. (2014). Study of the Association Between Hip Fracture and Acid‐Suppressive Drug Use in a UK Primary Care Setting. Pharmacotherapy: The Journal of Human Pharmacology and Drug Therapy. 34(6), 570-581. 10.1002/phar.1410

GINGOLD‐BELFER, R., BELOOSESKY, Y., AMARA, A., SHARON, E., BOLTIN, D., KOREN‐MORAG, N., et al. (2023). Different effects of chronic omeprazole use on osteoporotic fractures rate in the elderly. British Journal of Clinical Pharmacology. 89(12), 3539-3550. 10.1111/bcp.15847

LABENZ, C., WöRNS, M. A., ADARKWAH, C. C., GALLE, P. R., SCHATTENBERG, J. M. & KOSTEV, K. (2020). Proton pump inhibitors increase risk of bone fractures in men with cirrhosis: a population‐based study. Alimentary Pharmacology & Therapeutics. 52(6), 1042-1050. 10.1111/apt.16008

LEE, J., YOUN, K., CHOI, N.-K., LEE, J.-H., KANG, D., SONG, H.-J., et al. (2013). A population-based case–control study: proton pump inhibition and risk of hip fracture by use of bisphosphonate. Journal of Gastroenterology. 48(9), 1016-1022. 10.1007/s00535-012-0722-9

REYES, C., FORMIGA, F., CODERCH, M., HOYO, J., FERRIZ, G., CASANOVAS, J., et al. (2013). Use of proton pump inhibitors and risk of fragility hip fracture in a Mediterranean region. Bone. 52(2), 557-561. 10.1016/j.bone.2012.09.028

ROUX, C., BRIOT, K., GOSSEC, L., KOLTA, S., BLENK, T., FELSENBERG, D., et al. (2008). Increase in Vertebral Fracture Risk in Postmenopausal Women Using Omeprazole. Calcified Tissue International. 84(1), 13-19. 10.1007/s00223-008-9188-4

TORVINEN‐KIISKINEN, S., TOLPPANEN, A. M., KOPONEN, M., TANSKANEN, A., TIIHONEN, J., HARTIKAINEN, S., et al. (2018). Proton pump inhibitor use and risk of hip fractures among community‐dwelling persons with Alzheimer's disease—a nested case‐control study. Alimentary Pharmacology & Therapeutics. 47(8), 1135-1142. 10.1111/apt.14589

VAN DER HOORN, M. M. C., TETT, S. E., DE VRIES, O. J., DOBSON, A. J. & PEETERS, G. M. E. E. (2015). The effect of dose and type of proton pump inhibitor use on risk of fractures and osteoporosis treatment in older Australian women: A prospective cohort study. Bone. 81675-682. 10.1016/j.bone.2015.08.024
